# Unveiling the role of hexon-associated host proteins in fowl adenovirus serotype 4 replication

**DOI:** 10.3389/fvets.2025.1562872

**Published:** 2025-06-03

**Authors:** Xiaojun Zhang, Xueping Wang, Yina Zhao, Shifan Li, Xingang Xu

**Affiliations:** ^1^College of Veterinary Medicine, Northwest A&F University, Yangling, China; ^2^College of Animal Science and Technology, Tarim University, Alaer, China

**Keywords:** fowl adenovirus serotype 4, hexon protein, CCT5, tandem affinity purification, bioinformatics analysis, interaction protein

## Abstract

Fowl adenovirus serotype 4 (FAdV-4), a member of the family Adenoviridae and the genus *Aviadenovirus*, is responsible for a significant number of emerging diseases that cause substantial economic losses in the poultry industry worldwide. The hexon protein plays a crucial role in inducing autophagy and apoptosis and promoting virus replication. Identifying host factors that interact with the hexon protein is essential for elucidating the pathogenesis of FAdV-4. In this study, tandem affinity purification followed by mass spectrometry (TAP/MS) was used to screen the interacting proteins of hexon in leghorn male hepatoma (LMH) cells for the first time. A total of 82 hexon-associated proteins were identified in LMH cells expressing hexon compared with cells expressing the empty vector (NC). Gene Ontology and Ingenuity Pathway Analysis provided functional annotations of the hexon-interacting proteins and revealed that these proteins were associated with multiple biological functions, including virus infection, the cell cycle, endocytosis and the phagosome. Western blot and coimmunoprecipitation validation tests revealed that randomly selected significant proteins (CCT5, CCT7, and HSP70) interact with hexon, and these results are consistent with those of TAP/MS. Among them, the overexpression of CCT5 inhibited virus replication, whereas blocking CCT5 increased virus replication. In conclusion, this study demonstrated the successful screening of host proteins interacting with the hexon protein. The findings of this study will lead to a better understanding of the molecular mechanisms of hexon, thus benefiting the development of effective antiviral strategies.

## 1 Introduction

Hydropericardium hepatitis syndrome (HHS) caused by highly pathogenic fowl adenovirus serotype 4 (FAdV-4) is a severe infectious disease that kills poultry (1, 2). HHS frequently occurs and predominantly affects broilers, laying hens, and ducks between the ages of 3 and 5 weeks, with an onset period typically ranging from 7 to 10 days (3). Mortality peaks between 3 and 7 days following the onset of HHS and is characterized by rapid viremia and extensive pathological damage across multiple organs (4). Since the first outbreak of HHS in the Ankara region of Pakistan, the disease has subsequently disseminated globally (5). Prior to 2014, occurrences of HHS in domestic chicken flocks in China were sporadic. However, beginning in 2015, outbreaks of HHS were reported in Fujian, Shaanxi, Henan, Hebei, Shandong, Anhui, Liaoning, and other provinces and regions, resulting in significant economic losses to China’s domestic poultry industry, with a high mortality rate of 80% in infected chickens (6, 7).

The FAdV-4 virion is characterized as an icosahedral, nonenveloped virus with a double-stranded linear DNA genome, measuring 70 ~ 90 nm in diameter and possessing a genome length of 36 kilobases (8). The principal structural proteins constituting the FAdV-4 capsid include hexon, penton, and fiber proteins, with hexon serving as the predominant structural component of the adenovirus capsid (9). The hexon gene is 2,730 bp in length and encodes a protein of 910 amino acids, with an approximate molecular weight of 120 kilodaltons (10). The hexon protein is integral to viral invasion, facilitating virus entry into the nucleus, contributing to the replication of late viral genes, and inducing viral apoptosis and autophagy (8, 11, 12). Recent findings indicate that hexon is intimately associated with the pathogenicity of avian adenovirus and interacts with the molecular chaperone chaperonin-containing TCP-1 subunit 7 (CCT7) during FAdV-4 infection, thereby regulating potential targets of FAdV-4 infection (13). Hexon has also been shown to interact with various members of the heat shock protein family, including HSPA8, HSP70, and HSPD1, to modulate the replication of FAdV-4 (14). Furthermore, the hexon protein has been identified as a pivotal virulence factor in FAdV-4 (10, 15, 16). It is known that infectious diseases develop due to the complicated interactions between pathogens and their hosts. The hexon protein plays multiple roles in the FAdV-4 life cycle, but research on host factors regulating FAdV-4 infection remains relatively rare.

In this study, tandem affinity purification technology combined with mass spectrometry (TAP/MS) was utilized to screen potential host factors interacting with the FAdV-4 hexon protein. A total of 82 host proteins interacting with hexon were identified, and six potential TCP-1 ring complex/chaperonin-containing TCP-1 (TRiC/CCT) subunits were notably enriched. Among these subunits, CCT5 was found for the first time to inhibit FAdV-4 replication in LMH cells. These results demonstrate the important role of CCT5 as a host-interacting factor with the FAdV-4 hexon protein and provide a foundation for research on the pathogenic mechanism of FAdV-4 and its antiviral effects.

## 2 Materials and methods

### 2.1 Cells, viruses, antibodies, and reagents

The Leghorn male hepatoma (LMH) cell line was maintained in our laboratory, while the human embryonic kidney cell line 293 (HEK293) was procured from the American Type Culture Collection (ATCC). Additional reagents utilized in this study included the pLVX-Puro-MCS-3flag-TST lentiviral expression vector (FitGene, China; catalog no. 632168), pLVX-Puro-3flag-N-EGFP (FitGene, China; catalog no. FT120), pLVX-shRNA2-Puro (FitGene, China; catalog no. FT215), lentivirus packaging plasmid (FitGene, China; catalog no. 12258), *Escherichia coli* DH5α competent cells (TianGen, China; catalog no. CB101-02), Agarose Gel DNA Recovery Kit (Sanggon, China; catalog no. B518131), restriction enzymes (Thermo Scientific, USA; *NdeI*, ER0581; *XbaI*, FD0685), Plasmid Extraction Kit (Sanggon, China; B518191-0050), Prime Premix (Takara, Japan; R045A), ClonExpress II One Step Cloning Kit ligase (Vazyme, China; C112-2), DNA Gel Recovery Kit (Takara, Japan; 9762), DNA Purification Kit (Takara, Dalian, China; D1300), Reverse Transcription Kit (Qiagen, Germany; 218061), 250 bp DNA Ladder (TSINGKE, China; TSJ105-100), DL15000 DNA Marker (Yuanye, China; S28584), Protein Marker (Solarbio, China; PR1910), BCA Protein Quantification Kit (Life Technology, USA; 23225), and fetal bovine serum (Gibco, USA; 10500064). Antibodies specific for CCT5 (catalog no. 11603-1-AP), CCT7 (catalog no. 15994-1-AP), and HSP70 (catalog no. 10995-1-AP) were procured from Proteintech, China. The anti-FLAG antibody (catalog no. F1804) was obtained from Sigma, USA, and the Twin-Strep-tag (TST, catalog no. FI01110S) was sourced from FitGene, China. Anti-FLAG magnetic beads (catalog no. M8823) were also acquired from Sigma, USA. Additionally, goat anti-mouse IgG (H + L) (catalog no. HS201) and goat anti-rabbit IgG (H + L) (catalog no. HS101) were purchased from TransGen, China.

### 2.2 Construction of LMH cells expressing hexon and CCT5

The sequences of FAdV-4 hexon gene (accession no. KU877429.1) and CCT5 gene (accession no: A0A3Q2UA96) were chemically synthesized by Sangon Biotech. The hexon gene fragment was subsequently ligated into the cleavage site between *NdeI* and *XbaI* of the pLVX-Puro-MCS-3flag-TST vector. Similarly, the CCT5 gene fragment was ligated into the cleavage site between *MluI* and *BamHI* of the pLVX-Puro-3flag-N-EGFP vector. The recombinant plasmids pLVX-Puro-hexon-3flag-TST (Flag-TST-Hexon) and pLVX-Puro-3flag-N-CCT5-EGFP (EGFP-CCT5-3flag) were transformed, and positive clones were selected for verification and identification by sequencing.

Recombinant lentiviruses were produced by cotransfecting HEK293 cells with the lentiviral expression plasmid (pLVX-Puro-hexon-3flag-TST or pLVX-Puro-3flag-N-CCT5-EGFP) and the packaging plasmid Fitvirus Mix (pGag/Pol, Rev. and VSV-G) using the Fitvirus Lentivirus Packaging Reagent (Junhui, Guangzhou, China) according to the manufacturer’s instructions, while an empty vector was used as a negative control. The LMH cells were subsequently infected with lentiviruses expressing hexon and then selected with 2 μg/ml puromycin (Thermo Fisher, USA). Total cellular proteins were extracted from the resulting LMH cells, and Western blot analysis was conducted using an anti-FLAG antibody to detect protein expression. All the primers used are listed in Table 1.

**Table 1:** Primers used in this study.

Primers	Sequence (5′→3′)
Hexon-R	5′TACCGGACTCAGATCTCGAGATGGCGGCCCTCACGCCC3′
Hexon-F	5′GTCAGATCCCATGGATCCCACGGCGTTGCCTGTGGC3′
CCT5-R	5′GGTCTTTGTAGTCGGATCCCTCTTCAGATTCTCCAGGC33′
CCT5-F	5′CTGTACAAGGGCGGTTCATCGGCCATGGGGACGCTG 3′
siCCT5-1	5′-GGAACAGGCTGAACAATTACT-3′
siCCT5-2	5′-GCTGTGAATGCTGTACTGACA-3′
siCCT5-3	5′-GGGAGTGATCGTGGATAAAGA-3′
Hexon mRNA –R	5′-GAACATCCCTTGGGCCCA-3′
Hexon mRNA --F	5′-CGGAGTCGTGATACAGCA3′
β-actin-R	5′-CTCTATCCTGGCCTCCCTGT-3′
β-actin-F	5′-GCTGACACCTTCACCATTCC-3′

### 2.3 RNA interference

For the CCT5 gene, three pairs of short hairpin RNA (shRNA) constructs and one pair of negative control sequences were constructed and synthesized by Sangon Biotech, as shown in Table 1. These fragments were subsequently ligated into the pLVX-shRNA2-Puro vector. CCT5-shRNA plasmids were transfected into HEK293 cells. After a 72-h incubation period, the supernatant was harvested, centrifuged at 12,000 × *g* for 10 min and added to LMH cells, after which 2 μg/ml puromycin was used for selection of the target cells. After screening, CCT5 protein and mRNA expression levels were determined by Western blotting and qPCR, respectively.

### 2.4 Tandem affinity purification

The LMH cells were scraped off the dishes with a cell scraper, transferred to 1.5-ml EP tubes, and centrifuged at 1000 × *g* for 5 min. The LMH cells were washed three times with phosphate-buffered saline (PBS), and the supernatant was discarded each time. Next, 1 ml of precooled lysis solution (155 mM ammonium chloride, 10 mM potassium bicarbonate, 0.1 mM Na2-EDTA, pH = 7.0) was added to each 1.5-ml EP tube, and the cells were lysed on ice for 10 min. The lysate was subsequently centrifuged (12,000 × *g*, 15 min, 4°C), the supernatant was collected, and 50 μl from each tube was used as the corresponding input to transfer the supernatant into prewashed TST resin, which was subsequently incubated at 4°C in a mixer for 4 h. After the incubation period, the column was removed, and the effluent was collected. The column was then rinsed with the previous lysis buffer, with 1 ml used for each rinse, for a total of three rinses. Following rinsing, the samples were eluted with biotin, and the TST eluate was obtained, of which 50 μl was saved (for subsequent detection). An appropriate amount of anti-FLAG magnetic beads were washed three times with PBST and incubated with the cell lysis supernatant at 4°C overnight. The beads were then washed three times with 1 ml of cell lysis buffer and eluted with 3 × FLAG peptide for 10 min. The resulting mixture in a 1.5-ml centrifuge tube was immediately centrifuged at low speed and placed in a magnetic rack to separate the magnetic beads and liquid, and the liquid was transferred into a new 1.5-ml EP tube. The product was divided into two parts: one part was mixed in loading buffer, boiled in a water bath for 10 min, centrifuged at 12,000 × *g* at 4°C for 3 min and used for Western blotting; the other portion was directly digested by enzymes for mass spectrometry (MS).

### 2.5 Mass spectrometry analysis

LMH cells expressing the flag empty vector or flag-TST-hexon were purified using TAP technology and then sent to Huijun Biological Company for MS analysis. The raw MS data were processed and converted into a mascot generic format (MGF) file using Proteome Discoverer 1.4 (Thermo Fisher Scientific). The MGF file and protein retrieval library were input into ProteinPilot Software 4.5 (version 1,656, AB Sciex) for MS retrieval. All the identified proteins were classified based on their functional annotation using the Gene Ontology (GO, http://www.geneontology.org) analysis tool in the Database for Annotation, Visualization and Integrated Discovery (DAVID) version 6.8. Pathway analyses were performed to elucidate the significant pathways of proteins according to the Kyoto Encyclopedia of Genes and Genomes (KEGG) pathway database.^1^ Analysis of the protein interaction networks to identify target proteins was conducted using the STRING protein–protein interaction (PPI) network function enrichment analysis tool.^2^ All analyses were carried out with three technical and biological replicates.

### 2.6 Coimmunoprecipitation (Co-IP) assays and Western blot analysis

Flag-TST-Hexon-overexpressing LMH cells and Flag-TST-overexpressing LMH cells were detached with a cell scraper, transferred to a 1.5-ml EP tube containing a precooled equal volume of cell lysis buffer (Beyotime, Shanghai, China), and lysed by incubation on ice for 5 min. After centrifugation, 50 μl of supernatant was retained as the input sample, and the rest was subjected to Co-IP. Then, 30 μl of anti-FLAG magnetic beads were washed three times with cell lysis buffer. The supernatants of the LMH cells stably transfected with the flag control vector were used as the control group, and the LMH cells stably expressing the flag-TST-hexon protein were used as the experimental group. They were added to the magnetic beads in separate tubes and incubated at 4°C overnight. The FLAG magnetic beads were subsequently placed on a magnetic separator, the supernatant was discarded, the beads were washed three times with lysis buffer, and then FLAG elution buffer was added and incubated for 15 min. The magnetic beads and liquid were separated with a magnetic separator, and the resulting eluate was transferred into a new 1.5-ml EP tube. The mixture was transferred into a new 1.5-ml EP tube to collect the eluent. A total of 10 μl of each Co-IP product was mixed with loading buffer, boiled for 10 min, and centrifuged at 1,200 × *g* and 4°C for 3 min. Following these steps, Western blot analysis was performed following standard principles (17). The following primary antibodies were used: anti-flag (1:1,000), anti-CCT5 (1:2,000), anti-CCT7 (1:1,000), and anti-HSP70 (1:2000). The secondary antibodies used were goat anti-mouse IgG (H + L) (1:2,000) and goat anti-rabbit IgG (H + L) (1:2,000).

### 2.7 Real-time quantitative polymerase chain reaction (qPCR)

Total RNA was extracted from LMH cells using TRIzol reagent (Vazyme) according to the instructions provided by the manufacturer. The extracted total cell RNA was quantified at 1 μg and reverse-transcribed with a reverse transcription kit (Vazyme). A Roche fluorescence quantitative PCR system was used, and quantitative polymerase chain reaction (qPCR) was conducted with 2 × SYBR Green Master Mix. The mRNA expression levels of hexon and CCT5 were quantitatively analyzed by a relative quantitative method. The sequences of primers used for qPCR are listed in Table 1.

### 2.8 Statistical analysis

All experiments were performed at least 3 times, and the results were analyzed by using Student’s t test. Significant differences were determined as *p < 0.05.

## 3 Results

### 3.1 Construction of LMH cells expressing hexon and CCT5

**Figure 1:**
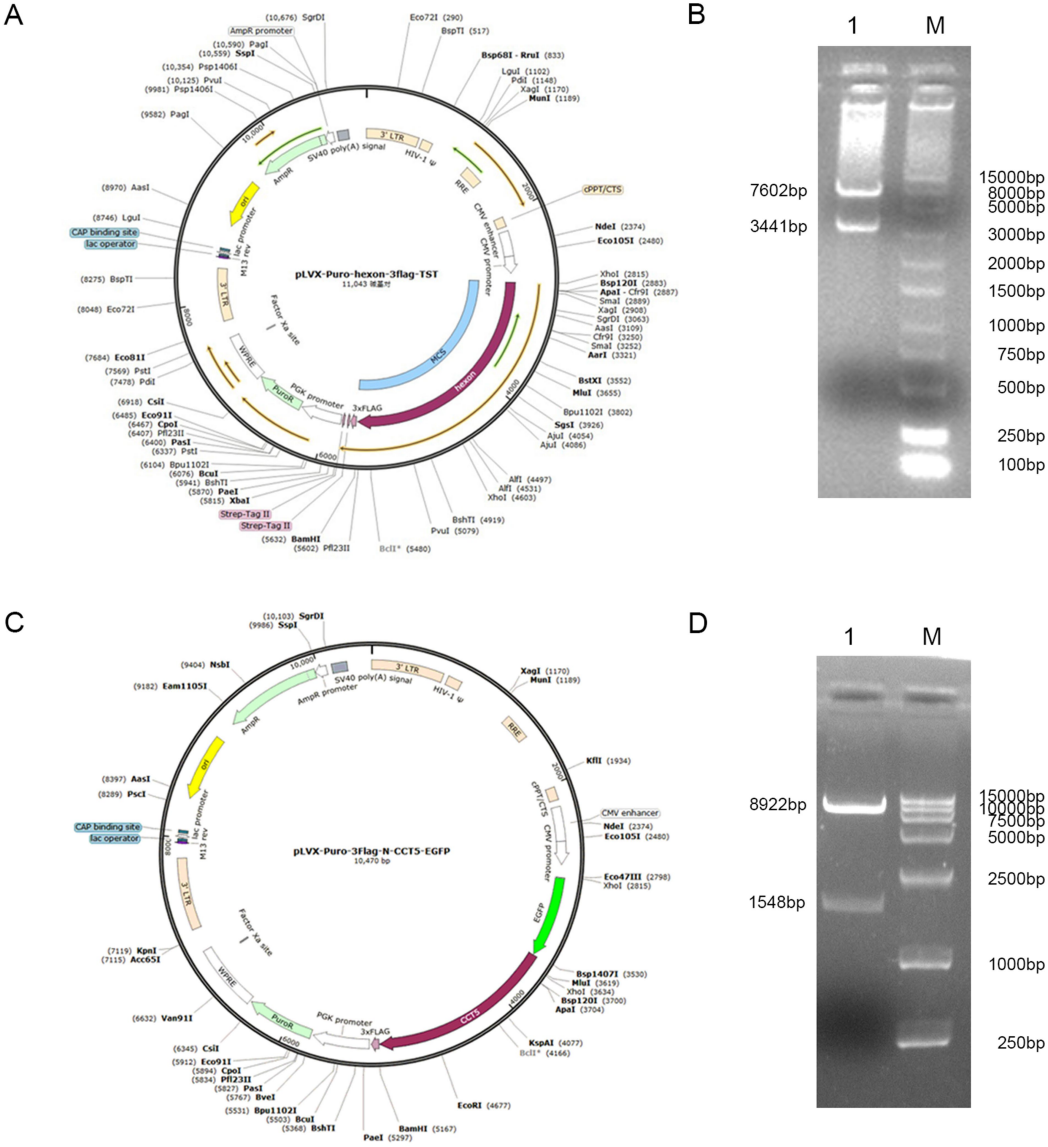
Recombinant vector identified by enzyme digestion. **(A)** Lentiviral vector of pLVX-Puro-hexon-3flag-TST; **(B)** The restriction enzyme digestion product of pLVX-Puro-hexon-3flag-TST using *NdeI/XbaI* (Lane 1); 250 bp DNA Ladder (Lane M); **(C)** Lentiviral vector of pLVX-Puro-3flag-N-CCT5-EGFP; **(D)** The restriction enzyme digestion product of pLVX-Puro-3flag-N-CCT5-EGFP using *MluI*/*BamHI* (Lane 1); DL15000 DNA Marker (Lane M).

The double-labeled lentivirus expression vectors pLVX-Puro-hexon-3flag-TST and pLVX-Puro-3flag-N-CCT5-EGFP were double-digested with *NdeI*/*XbaI* and *MluI*/*BamHI*, respectively. The products were identified by 1.0% agarose gel electrophoresis, and the results revealed approximately 3.4-, 7.6-, 1.5-, and 8.9-kb fragments in the products digested by *NdeI/XbaI* and *MluI*/*BamHI* (Figures 1A–D). The sequencing results confirmed that the constructed vector was correct (Supplementary file S1).

### 3.2 Identification of hexon-interacting factors from LMH cells

**Figure 2:**
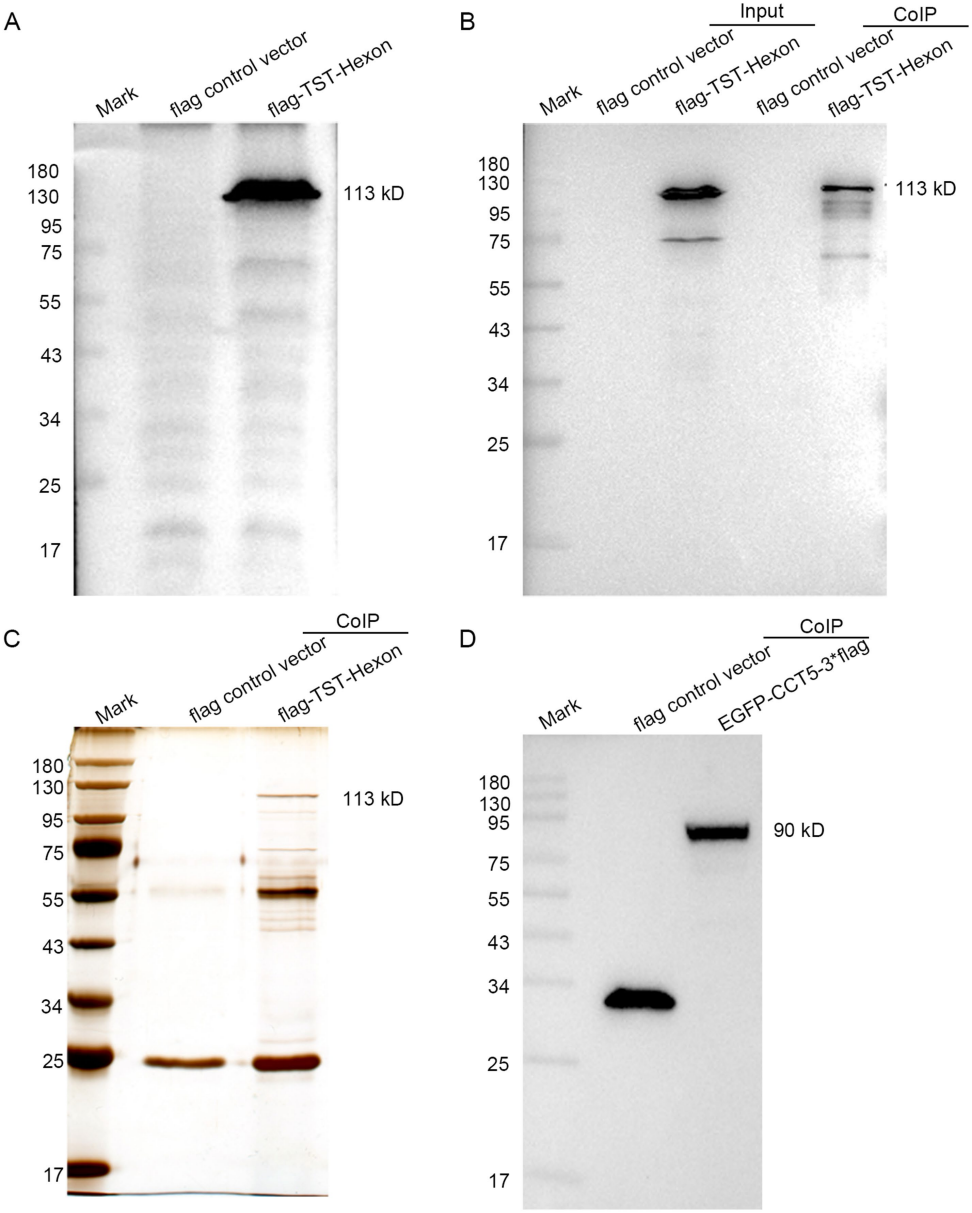
Confirmation of LMH cells expressing hexon and CCT5. **(A)** pLVX-Puro-3flag-TST and pLVX-Puro-hexon-3flag-TST were detected by Western blot; **(B)** Hexon proteins were identified by anti-TST antibody; **(C)** Silver stain gel of pLVX-Puro-3flag-TST (control vector) and silver stain gel of pLVX-puro-hexon-3flag-TST tandem affinity-purified from LMH cells; **(D)** EGFP-3flag and EGFP-CCT5-3flag were detected by Western blot.

The Flag-TST-hexon lentivirus was transduced into LMH cells. The Western blot results indicated the absence of flag-TST-hexon protein bands in the control transduced strain. However, specific flag-TST-hexon protein bands near 113 kDa were detected in the overexpressed transfected strain. These findings confirmed the successful construction of the stably overexpressing strain harboring the hexon gene (Figure 2A). The resulting TAP products were then analyzed by Western blotting with anti-TST antibodies. The results revealed that the hexon protein could be detected in both the input and the TAP eluent groups expressing the flag-TST-hexon protein but not in the flag control vector group (Figure 2B). This indicated that the TAP process was successful. The proteins that interacted with hexon were then obtained by SDS-PAGE and silver stained with the eluate from TAP (Figure 2C). EGFP-3flag (control vector) and EGFP-CCT5-3flag were transfected into LMH cells. The Western blot results indicated that specific EGFP-CCT5-3flag protein bands near 90 kDa were detected in the overexpressed transfected strain. These findings confirmed the successful construction of the overexpression strain harboring the CCT5 gene (Figure 2D).

The mass spectrometry results revealed that the flag empty vector was used as the control to eliminate nonspecific and miscellaneous proteins, resulting in the identification of 82 proteins interacting with hexon. Based on the potential functions of the proteins and their SAINT scores, we compiled a list of the most likely proteins that impact viral replication, as shown in Table 2. Additionally, all host-interacting proteins of hexon identified in chicken cells are included in Supplementary file S2 for reference.

**Table 2:** Hexon–host interacting proteins in chicken cells (selected proteins).

Uniprot ID	Protein name	Gene	Protein function
F1NFJ0	Minichromosome Maintenance Complex Component 3	MCM3	DNA replication
Q5ZKR8	Minichromosome Maintenance Complex Component 6	MCM6	DNA replication
P0CB50	Peroxiredoxin-1	PRDX1	NF-kappa-B regulation
E1BY89	60S ribosomal protein L23	RPL23	Translation
A0A1D5P3B1	60S ribosomal protein L11	RPL11	Translation
A0A1D5NVI1	60S ribosomal protein L9	RPL9	Translation
E1BU66	60S ribosomal protein L38	RPL38	Translation
Q5ZKX2	Proteasome 26S Subunit, ATPase 5	PSMC6	Proteasomesubunit
Q5ZLU4	26S proteasome non-ATPase regulatory subunit 2	PSMD2	Proteasomesubunit
R4GGJ0	Ribosomal Protein S16	RPS16	Viral mRNA translation
A0A1D5NZ06	Ribosomal Protein S27	RPS27	Viral mRNA translation
F1N9U0	Pre-mRNA-processing factor 6	PRPF6	Pre-mRNA processing
F1NV33	DNA mismatch repair protein Msh2	MSH2	DNA repair protein

### 3.3 Bioinformatics analysis of hexon-host interaction partners

**Figure 3:**
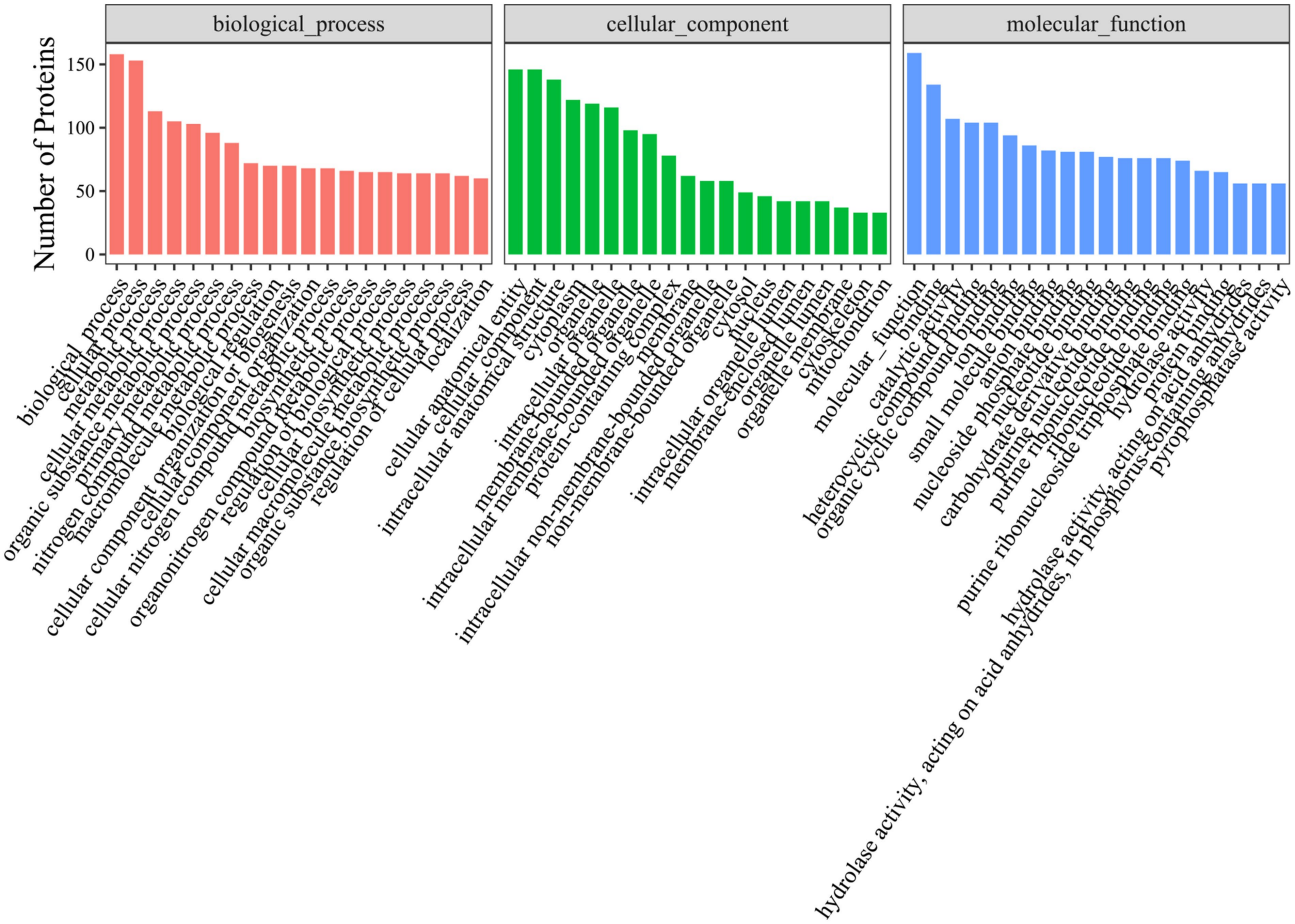
Gene Ontology (GO) terms enrichment analysis of hexon interacting proteins. Top 20 Gene Ontology (*p*-value < 0.05) analysis of relevant biological process, cellular components, and molecular functions. The x-axis represents functional groups, and the y-axis represents the number of proteins.

**Figure 4:**
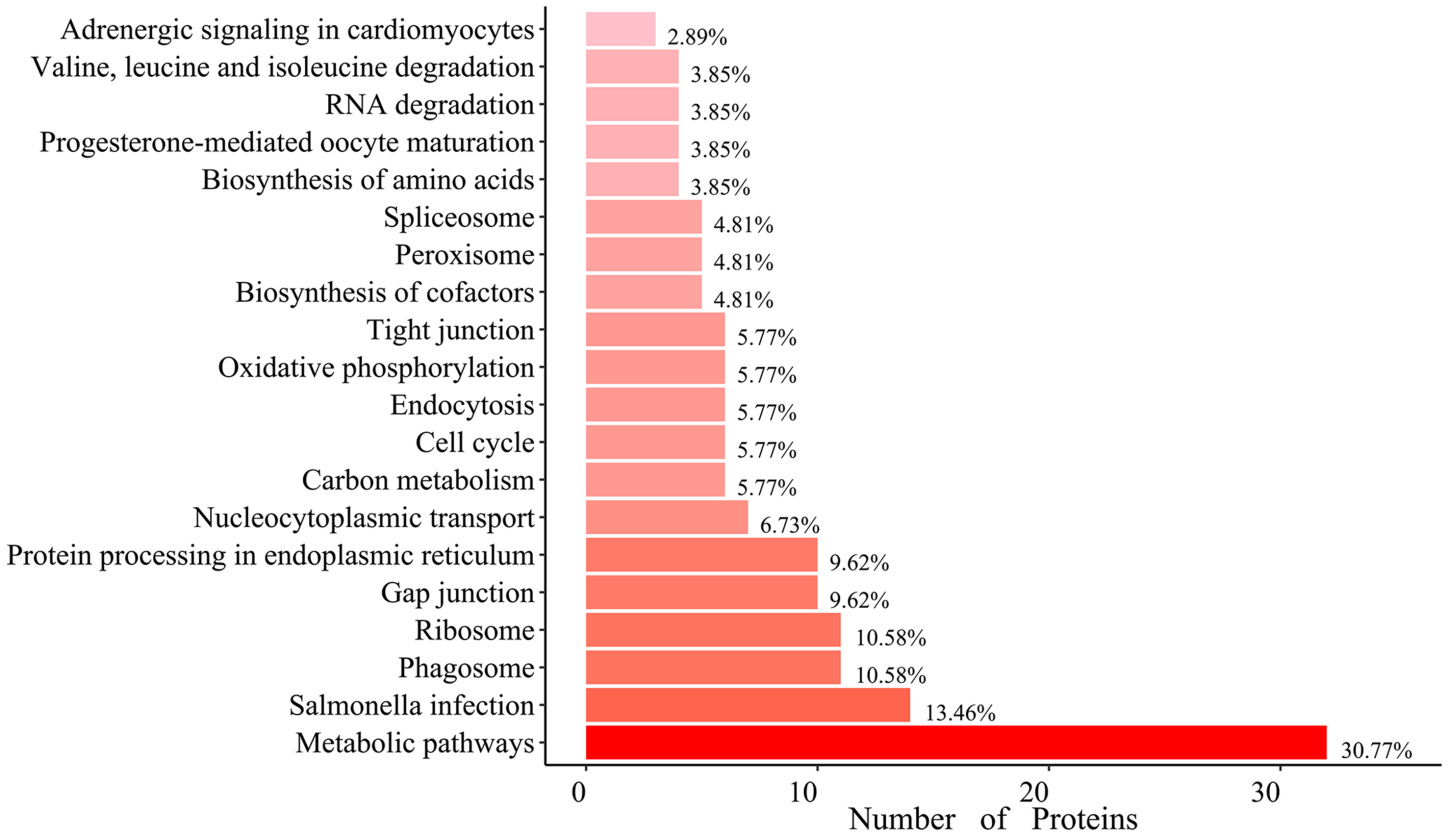
Enrichment of Kyoto Encyclopedia of Genes and Genomes (KEGG) pathway analysis of hexon interacting proteins. Top 20 KEGG pathways (*p*-value < 0.05) analysis of the relevant signal pathways. The x-axis represents the number of proteins, and the y-axis represents functional groups.

Gene Ontology (GO) analysis revealed that hexon-interacting proteins were significantly enriched in biological processes such as cell adhesion, glucose metabolism, and positive regulation of NF-κB transcription factor activity (Figure 3; Supplementary file S3). Kyoto Encyclopedia of Genes and Genomes (KEGG) analysis revealed that hexon-interacting proteins were significantly enriched in virus infection, the cell cycle, endocytosis and phagosome, and metabolic pathways, suggesting the potential roles of these proteins in FAdV-4 infection (Figure 4; Supplementary file S4).

**Figure 5:**
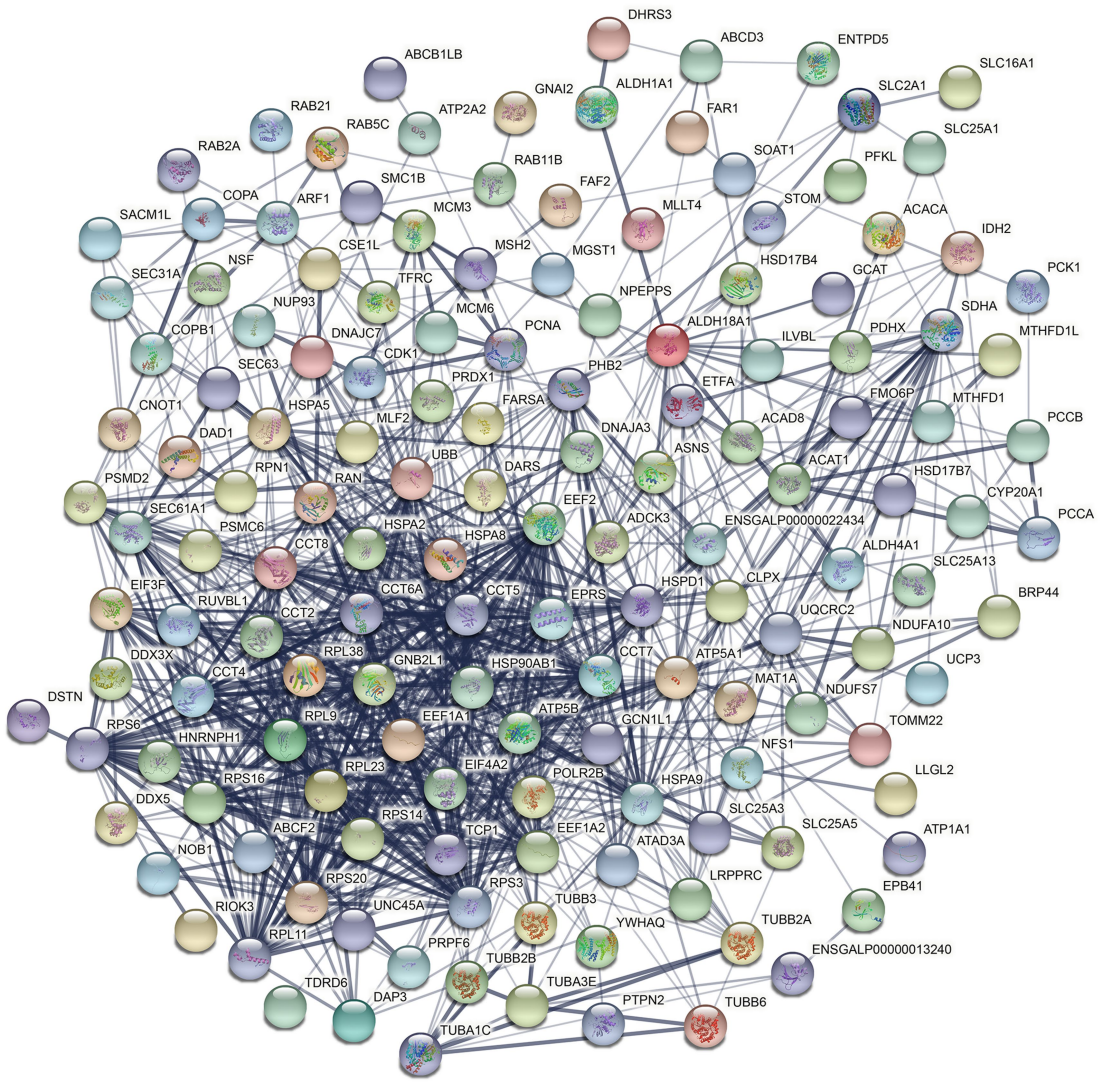
Construction of the PPI networks (Interconnected protein networks composed of differential proteins). The 82 core target interaction proteins were placed in the STRING protein network database to construct protein interaction networks. Nodes represent proteins. The node color represents the degree of expression difference of genes in the samples. In the protein association network diagram, the blue lines represent the connections between proteins, and the strength of the interaction confidence varies according to the depth of blue. The darker the color, the stronger the confidence.

To explore the potential functions of hexon in the interaction between FAdV-4 and host cells, we analyzed all the host proteins interacting with hexon using the STRING database and identified one potential cluster, which involves mainly ribosomal proteins, the heat shock protein (HSP) family, and the T-complex protein ring complex (TRiC) family (Figure 5; Supplementary file S5).

### 3.4 Identification of hexon and its interacting proteins by coimmunoprecipitation

**Figure 6:**
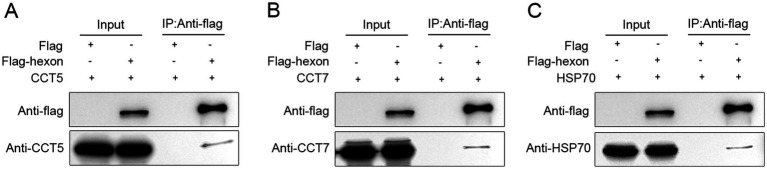
Identification of the interaction of the LMH cell proteins with the FAdV-4 hexon. Immunoprecipitation and western bolt were conducted to confirm the expression of bait and prey proteins in LMH cells **(A–C)**. Co-immunoprecipitated of hexon protein (113 kDa) with CCT5 (60 kDa), CCT7 (59 kDa), HSP70 (68 kDa). The cell lysates of the two groups (flag-TST-hexon and flag control vector) were incubated with anti-flag magnetic beads at 4°C overnight and then eluted with flag eluent to obtain Co-IP products. Co-IP products were boiled in loading buffer, and then the flag labeled CCT5, CCT7, HSP70 antibodies, or hexon specific antibodies were used for western bolt detection.

LMH cells transfected with the flag control vector were used as the control group, and LMH cells expressing the flag-TST-hexon protein were used as the experimental group. Co-IP of flag-TST-hexon protein (decoy protein) was conducted using anti-FLAG magnetic beads. In the detection results with the anti-FLAG antibody, the decoy protein could not be detected in the control group but was detected in the experimental group, indicating that the Co-IP experiment was successful (Figure 6A). In the detection results with the anti-CCT5 antibody, CCT5 could not be detected in the control group but was detected in the experimental group, indicating that there was an interaction between the hexon protein and CCT5 protein (Figure 6B). Similar results were obtained with the anti-CCT7 antibody and the anti-HSP70 antibody, indicating that the hexon proteins interact with the CCT7 protein (Figure 6C) and the HSP70 protein (Figure 6D), respectively.

### 3.5 Effects of CCT5 on FAdV-4 replication in host cells

**Figure 7:**
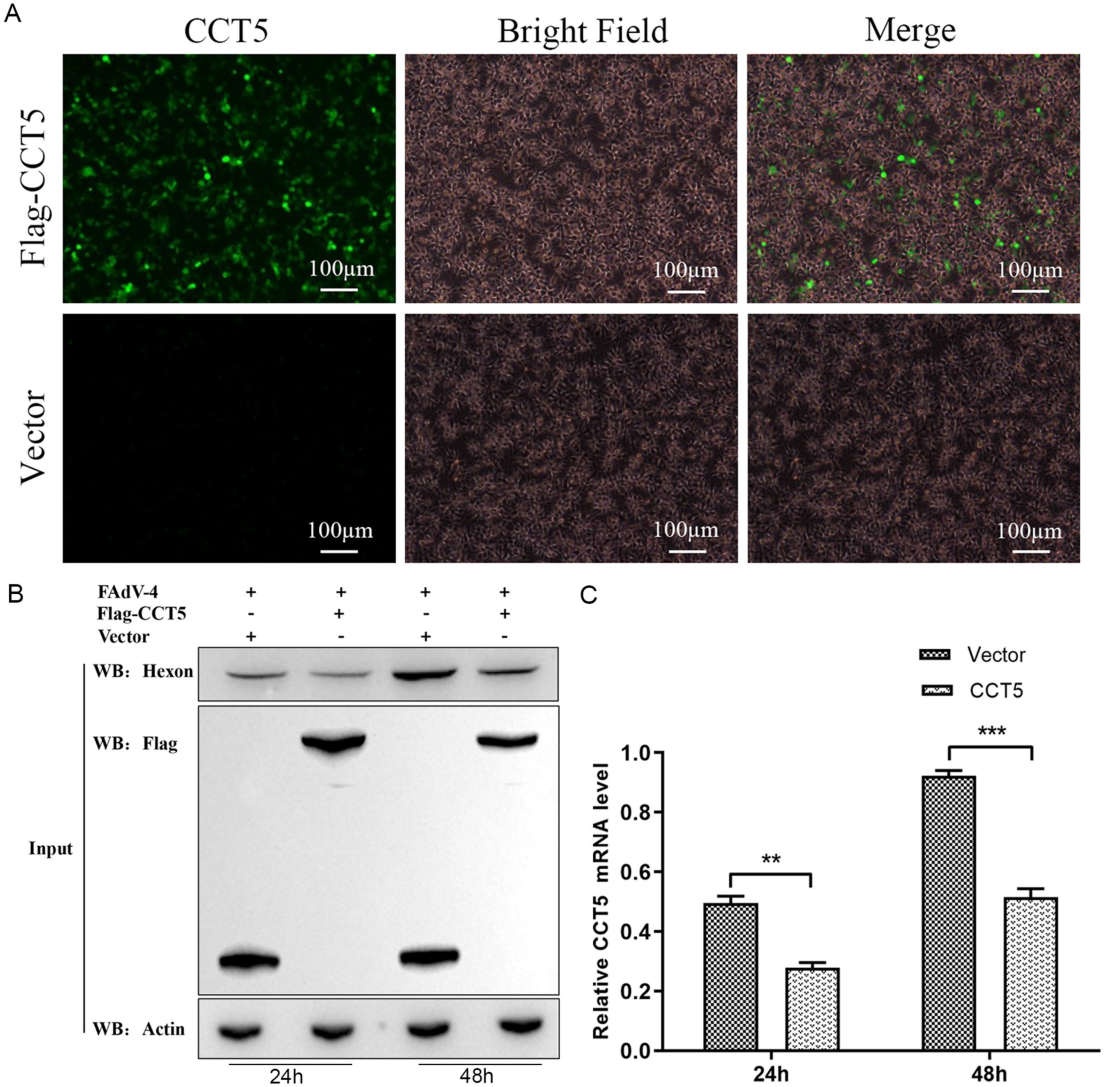
FAdV-4 infection inhibits the expression of CCT5 in LMH cell. **(A)** The overexpression of the flag-CCT5 vector and the flag control vector in LMH cells was assessed by microscopy; **(B)** LMH cells were infected with the indicated dose of FAdV-4 for 24 and 48 h. The protein levels of CCT5 and hexon were detected by Western blot; **(C)** LMH cells were infected with FAdV-4 for 48 h; the protein levels of CCT5 were detected by qPCR *Stands for *p* < 0.05, ** stands for *p* < 0.01. FAdV-4, Fowl adenovirus serotype 4; LMH, leghorn male hepatocellular.

To assess the impact of CCT5 on the replication of FAdV-4, the flag-CCT5 vector and flag control vector were overexpressed in LMH cells (Figure 7A). After a 24-h post-transfection period, the cell supernatant was removed, and the cells were subsequently infected with FAdV-4. Cell samples were collected at 24 and 48 h post-infection for Western blot and qPCR analyses. The results revealed a significant decrease in both hexon protein expression and mRNA levels in cells overexpressing CCT5 compared with those in the control group, indicating a suppressive effect on FAdV-4 replication (Figures 7B,C).

**Figure 8:**
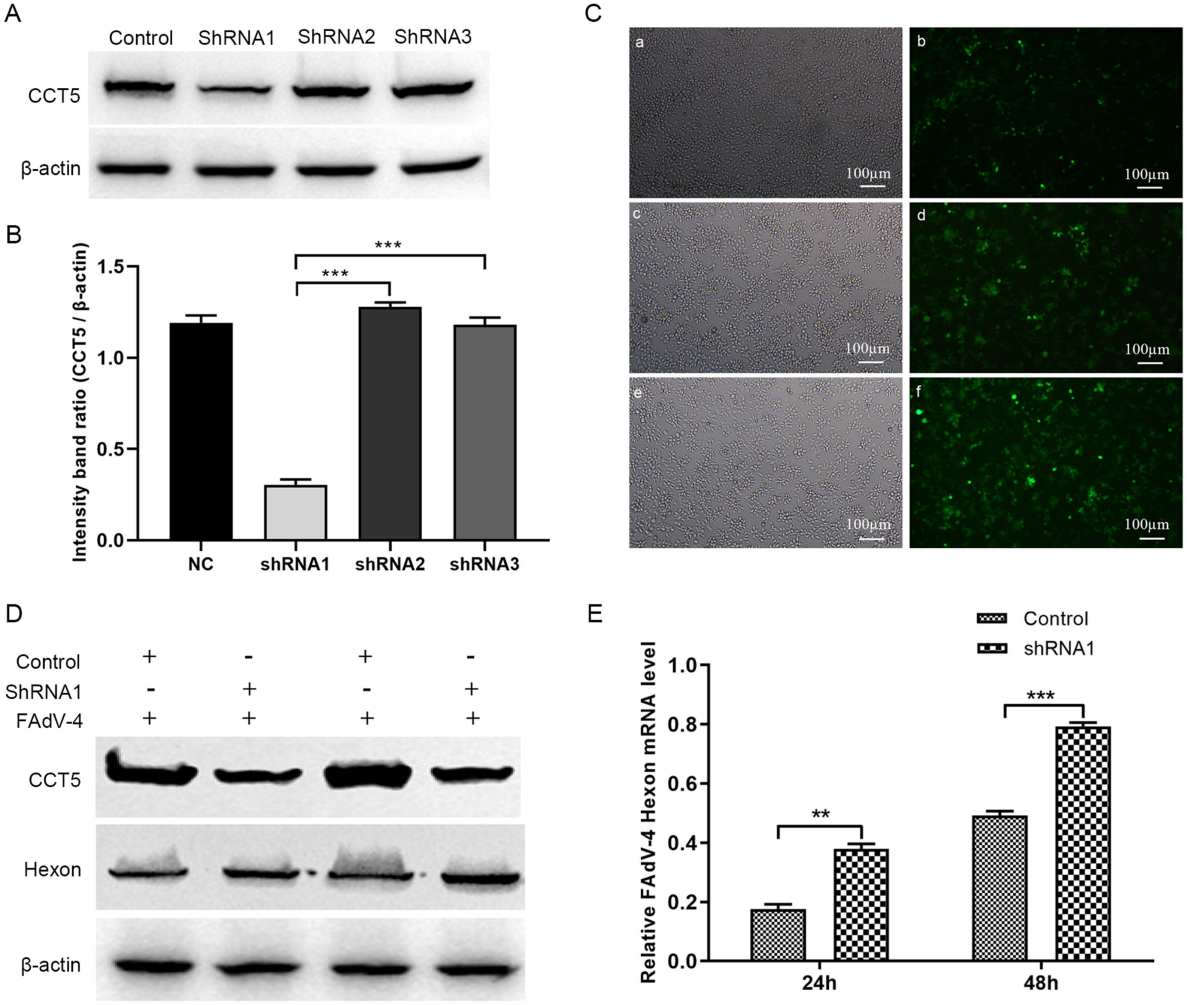
Knock down of CCT5 increased FAdV-4 replication. **(A)** Identification of CCT5-scilenced LMH cell line by Western-blot; **(B)** The relative CCT5 protein levels were estimated by histograms representing density readings of the gel bands, and the ratios were calculated relative to β-actin control; **(C)** Observation of CCT5-scilenced cell with a microscope; **(D)** Detection of FAdV-4 hexon protein expression by Western blot; **(E)** Detection of FAdV-4 hexon mRNA expression level by qPCR. *Stands for p < 0.05, **stands for p < 0.01. FAdV-4, Fowl adenovirus serotype 4; LMH, leghorn male hepatocellular.

To further elucidate the role of CCT5 in FAdV-4 replication, we knocked down CCT5 expression with short hairpin RNA (shRNA) (Figures 8A–C). CCT5-ShRNA1 was used in the following experiments. The stable CCT5-ShRNA1-knockdown cell line and CCT5-ShRNA1-control cell line were infected with FAdV-4 at a multiplicity of infection (MOI) of 0.1. The cell samples were collected at 24 and 48 h post-infection. Western blot and qPCR analyses revealed that the expression level of the FAdV-4 hexon protein was significantly higher in the CCT5-knockdown group than in the control group (Figures 8D,E). These findings clearly suggested that the downregulation of CCT5 could enhance the replication of FAdV-4.

## 4 Discussion

Hexon is the major protein of the FAdV-4 capsid and contains many neutralizing epitopes, especially neutralizing antigenic sites, which makes it the preferred protein for the development of diagnostic reagents and vaccines for FAdV-4 (18–20). Recent studies have shown that the hexon protein plays a crucial role in viral invasion, inducing viral apoptosis and autophagy (8, 11, 12). Moreover, the hexon gene is considered to be the key virulence gene for the enhanced pathogenicity of all novel FAdV-4 strains, whereas the activity of fiber 2 is limited to specific strains; hexon, not fiber 2, determines the pathogenicity of FAdV-4 (10). However, how hexon interacts with host proteins to participate in FAdV-4 replication remains to be elucidated. Studies on the biological functions and roles of the hexon protein are highly important for screening vaccines and antiviral drugs.

FAdV-4 is incapable of self-replication; during the FAdV-4 infection cycle, viral and host factors interact to advance the multistep process required to produce progeny virions. In this study, we first used MS to examine critical cellular proteins interacting with FAdV-4 hexon, and 82 host proteins were identified. GO enrichment analysis revealed four molecular functions (protein binding, protein transport, transmembrane transport and protein folding), three cellular functions (nucleotide binding, RNA binding and structural molecule activity), and five biological processes (cytoplasm, organelle membrane, endomembrane system, organelle envelope and viral transcription), all of which suggested the possible mechanism by which these genes might be involved in FAdV-4 pathogenesis. KEGG analysis revealed that these proteins were associated with multiple biological functions, including the cell cycle (e.g., PCNA, CDK1, SMC1A, MCM6, and MCM3), endocytosis and phagosome (e.g., SEC61A1 and F1NTM6), translation elongation factor (e.g., eEF1A1, EEF4A2, and eEF1A2), ribosomal protein (e.g., RPL9, RPL11, RPL14, RPL16, RPL23, RPS3, RPS6, RPS14, PRS16 and RPS20) and virus infection (e.g., TCP1, CCT2, CCT5, CCT6, CCT7, CCT8, HSP70, and HSP90), suggesting their potential significance in FAdV-4 infection.

Proliferating cell nuclear antigen (PCNA) is a 36-kDa nuclear protein that accumulates in the nucleus during the S phase of the cell cycle and is involved in DNA replication and DNA damage repair as a cofactor of DNA polymerase (21, 22). PCNA is a critical component of eukaryotic DNA repair, DNA replication, and chromatin assembly and is involved in regulating the cell cycle (23, 24). Previous studies have demonstrated that PCNA can interact with the PB2 protein of the influenza A virus, thereby inhibiting its nuclear import and subsequently suppressing the replication of the influenza A virus (22). PCNA can be transferred from the nucleus to the cytoplasm for stable expression after PRRSV infection. Silencing of PCNA can inhibit PRRSV replication, whereas its overexpression enhances virus replication. Therefore, PCNA is a potential target for preventing and controlling PRRSV infection (23). The SARS-CoV-2 M protein interacts with PCNA, affecting its movement to the nucleus to enhance viral replication (25). In addition, a previous study showed that PCNA is an adenovirus EIA-inducible factor that is intimately linked to adenovirus replication and cycle regulation. A PCNA promoter-binding protein, namely, ATF-1, RFXI or YYI, was subsequently shown to be involved in adenovirus replication (26, 27). In this study, hexon was found to interact with PCNA and may be involved in FAdV-4 virus replication, although the specific mechanism of action needs further exploration.

Eukaryotic protein translation elongation factor 1 (eEF1A) is a highly abundant cellular protein that is involved in protein degradation, apoptosis, heat shock, nucleocytoplasmic trafficking, and virus replication (28, 29). There is accumulating evidence that eEF1A is instrumental in the replication of different RNA and DNA viruses (30, 31). eEF1A is reported to be an antiviral host factor because it can reduce the replication efficiency of the virus by inhibiting the activity of the viral polymerase and the nuclear aggregation ability of the polymerase components (32). In addition, eEF1A1 plays a critical role in eukaryotes in avian reovirus sigma-C-induced apoptosis and the inhibition of avian reovirus growth (33). Therefore, we speculate that the hexon-interacting host protein eEF1A1 might be involved in the function of FAdV-4 infection, which needs further investigation.

Upon infecting a host cell, viruses initiate a program to produce numerous progeny. Although they employ various mechanisms to interact with the host, a crucial stage in their life cycle is the synthesis of viral proteins by the host’s ribosomes. It has been reported that ribosomal proteins (RPs) regulate transcription, translation, apoptosis, and virus replication (34–36). Interestingly, in addition to the regulation of viral infection by RPs, reports on their antiviral function have recently been published. The phosphorylation of RPS6 and translation of RP mRNA can inhibit host protein synthesis caused by poliovirus infection (37). RPL9 interacts with phosphoprotein P of rabies virus (RABV) and is translocated from the nucleus to the cytoplasm, where it inhibits RABV transcription (38). RPS20 inhibits the replication of classical swine 4 fever virus (CSFV) in cells by modulating Toll-like receptor 3 (TLR3), which can trigger the immune response (39). In this study, we screened ribosomal proteins that interact with hexon (Table 2). These identified RPs play important roles in the life cycle of various viruses, indicating their potential as targets for designing small-molecule antiviral drug treatments for HHS.

Importantly, numerous host proteins associated with the heat shock protein (HSP) family and the T-complex protein-ring complex (TRiC) family were identified through bioinformatics analysis. Heat shock proteins (HSPs) are a group of proteins that have been highly conserved throughout evolution and are essential to all living organisms (40). HSPs can be divided into six families according to their molecular weight, namely, small HSPs, HSP40, HSP60, HSP70, HSP90, and large HSPs (41). HSP70 members are located in numerous cellular components, such as the endoplasmic reticulum (HSPA5), the nucleus (HSPA1A and HSPA8), and mitochondria (HSPA9) (42). Heat shock 70-kDa protein 8 (HSPA8, Hsp70) is involved in viral entry, viral intracellular trafficking, virus disassembly, and viral genome replication (43–46). Recent studies on the interaction between Hsp70 and FAdV-4 have shown that Hsp70 cooperates with DnaJC7 to negatively regulate FAdV-4 replication by suppressing viral hexon (47). In addition, the cellular protein Hsp70 promotes FAdV-4 replication in LMH cells by interacting with the viral 100 k protein (14). However, it is not clear whether the Hsp70 protein participates in FAdV-4 invasion and viral genome replication during hexon protein interactions. Further functional analysis to explore the underlying mechanism is warranted.

Heat shock protein 90 (HSP90) is involved in diverse biological processes, such as virus infection, the immune response, and signal transduction (48–50). Many viruses depend on cellular HSP90 to complete their life cycles, with many especially dependent on the HSP90AA1 (HSP90α) and HSP90AB1 (HSP90β) isoforms (51, 52). HSP90AA1 is a multipathogen receptor that binds to dengue virus, lipopolysaccharide (LPS), and avibirnavirus (53). HSP90AA1 on the cell surface also recognizes the influenza A virus (IAV) subunit in the receptor-binding region of the IAV membrane protein hemagglutinin and thus initiates the autophagy pathway regulated by AKT–mTOR (51). The cell surface expression of HSP90AB1 is vital for recognition and binding by infectious bursal disease virus (IBDV) receptors to mediate the infection of cells (54). In addition, the cellular protein HSP90AB1 binds to the red-spotted grouper nervous necrosis virus (RGNNV) capsid protein and induces incomplete autophagy (55). HSP90AB1 may also promote viral replication by interacting with the classical swine fever virus (CSFV) protein NS5A-3, leading to autophagy activation through the mTOR signaling pathway (56). However, to date, there have been no reports on the functions of the host HSP90AB1 and HSP90AA1 proteins during FAdV-4 infection. Consequently, further studies on the role of the HSP90AB1 and HSP90AA1 proteins in regulating FAdV-4 infection during their interaction with hexon proteins are needed, which may provide new molecular targets for the development of antiviral drugs.

The T-complex protein ring complex (TRiC) is a large complex of 100–900 kDa formed by rings composed of eight different subunits (CCT1-CCT8), and these subunits are involved in various functions, such as cell proliferation, apoptosis, autophagy, and viral replication (57–59). In this study, we identified six potential TRiC protein subunits via MS (namely, TCP1, CCT2, CCT3, CCT5, CCT6, CCT7, and CCT8) that were able to bind the FAdV-4 hexon protein, suggesting that the hexon protein employs the TRiC/CCT complex as a chaperone during the replication of FAdV-4. Chaperonin-containing TCP1 subunit 5 (CCT5) belongs to the chaperone protein chaperonin (Cpn) family, functions in an ATP-dependent manner and can guide misfolded proteins to their natural state, which plays an important role in the maintenance of cell activity (60). Previous studies have shown that CCT5 also participates in the replication of several viruses (43–46). Here, one of the most important findings reported for the first time is the identification of CCT5 as a host-interacting factor with the FAdV-4 hexon protein. Compared with those in the control group, the expression levels of the hexon protein decreased in cells overexpressing CCT5, suggesting a suppressive effect on FAdV-4 replication. To further investigate the role of CCT5 in FAdV-4 replication, we employed Western blot and qPCR analyses, which revealed that the expression level of the FAdV-4 hexon protein was significantly greater in the CCT5-knockdown group than in the control group (Figures 8D,E). These findings indicate that the overexpression of CCT5 can inhibit FAdV-4 replication *in vitro*, suggesting that CCT5 may be a potential host factor against FAdV-4 infection. This discovery provides a foundation for further exploration of the pathogenic mechanism of FAdV-4 and antiviral research.

## Data Availability

The datasets presented in this study can be found in online repositories. The names of the repository/repositories and accession number(s) can be found in the article/Supplementary material.

## Footnotes

^1^ http://www.genome.jp/kegg

^2^ https://string-db.org
