# Supplementary material for: Unveiling the role of hexon-associated host proteins in fowl adenovirus serotype 4 replication
Source: Front Vet Sci. 2025 Jun 3;12:1562872. doi: 10.3389/fvets.2025.1562872 (PMC12170584; doi:10.3389/fvets.2025.1562872)

Supplementary file S1

Original sequencing peak mapping of pLVX-Puro-MCS-3flag-TST(+)-hexon

DH5a-JN-PEGFP-N-5-Premix-primer_A07：


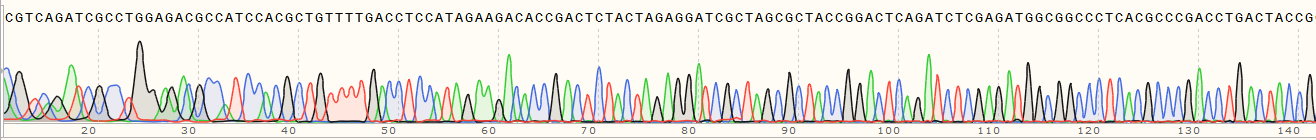


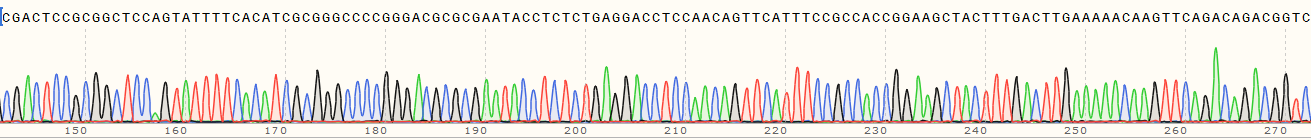


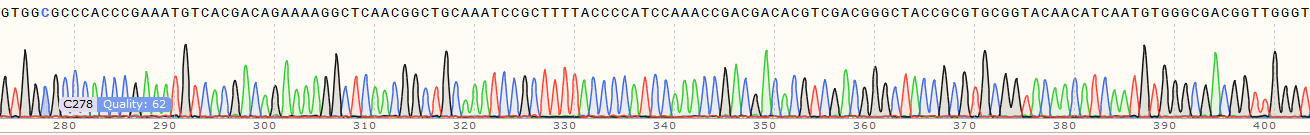


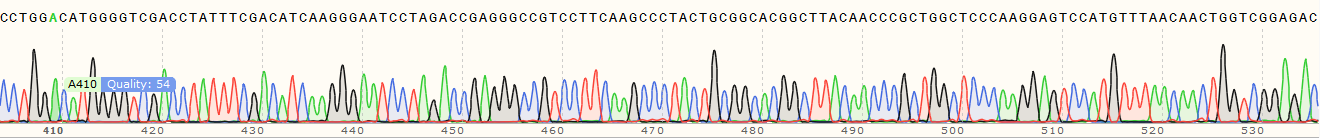


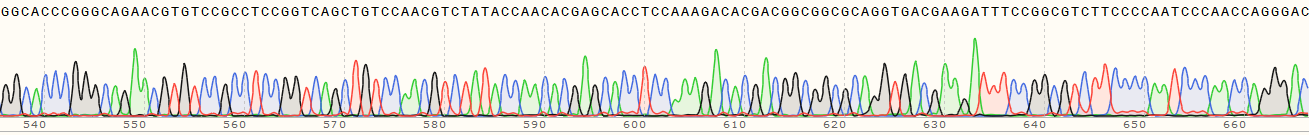


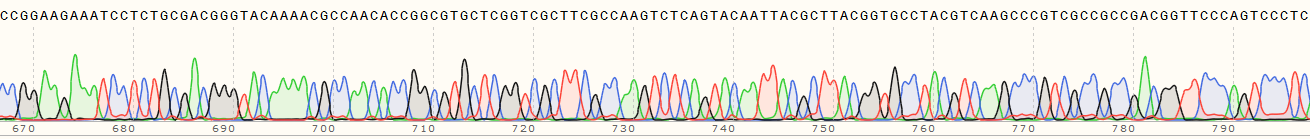


DH5a-JN-M-seq4868-Premix-primer_F09：


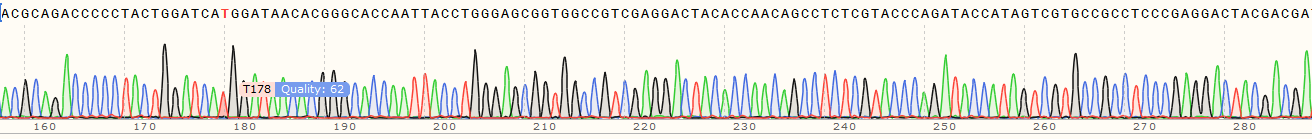


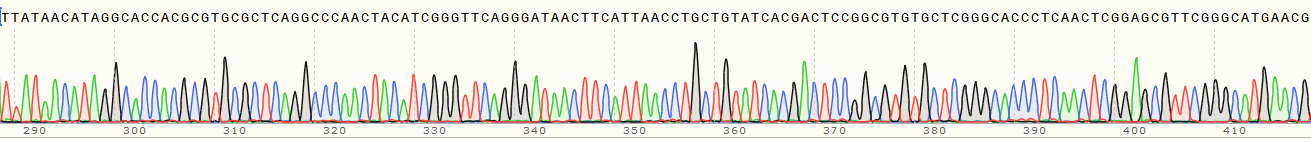


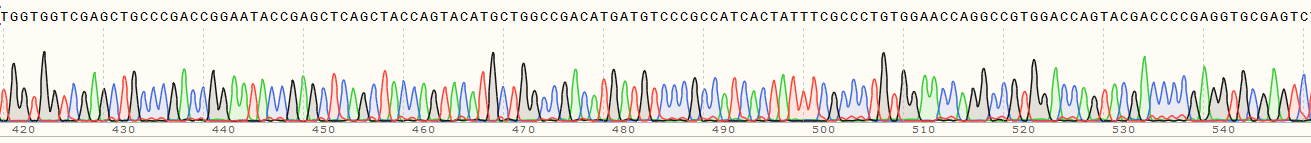


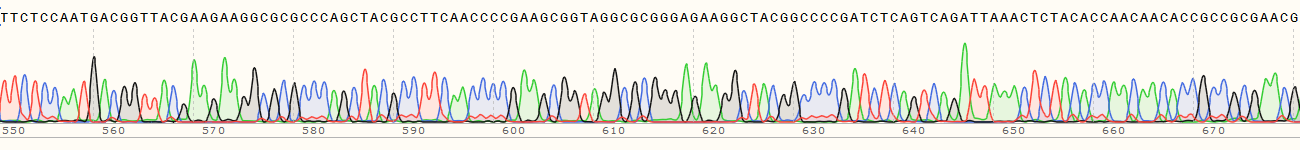

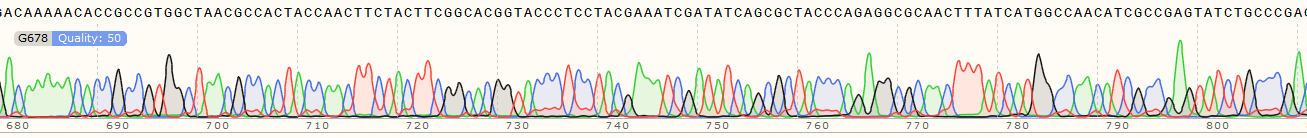


DH5a-JN-M-seq4869-Premix-primer_H10：


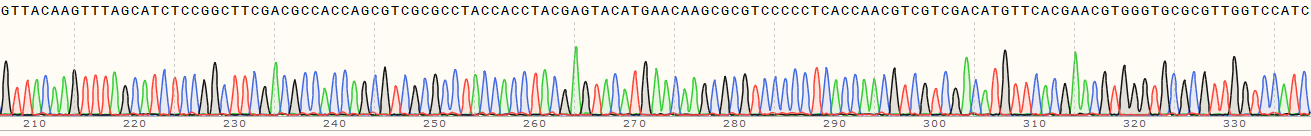


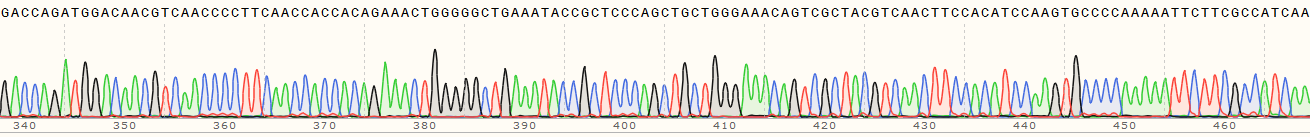


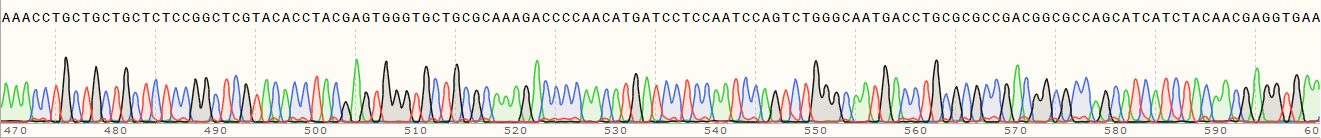


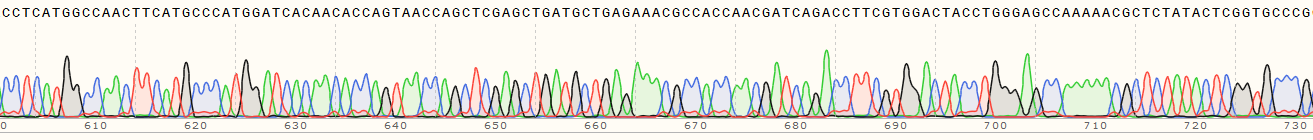


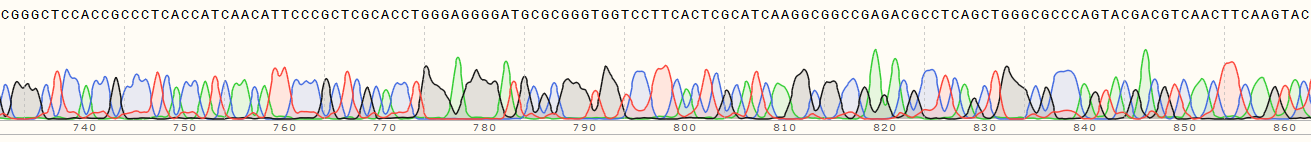


DH5a-JN-M-seq4870-Premix-primer_F11：


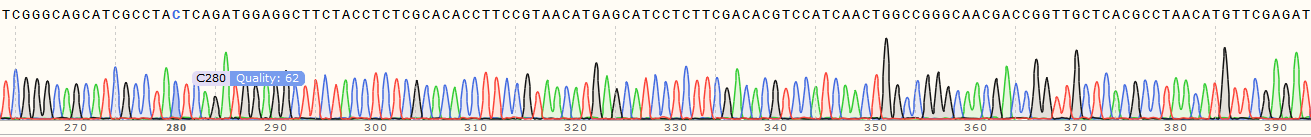


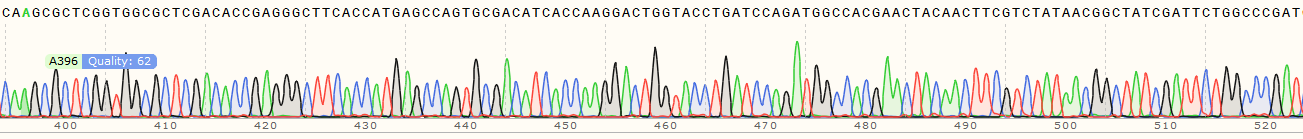


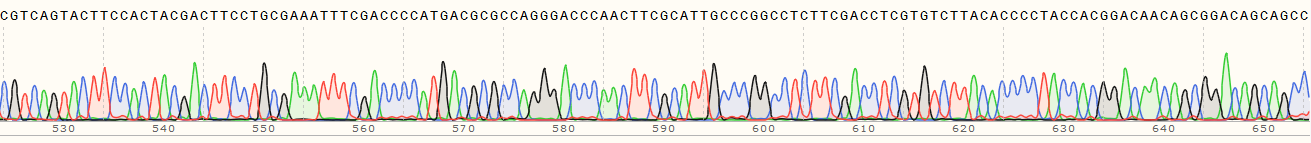


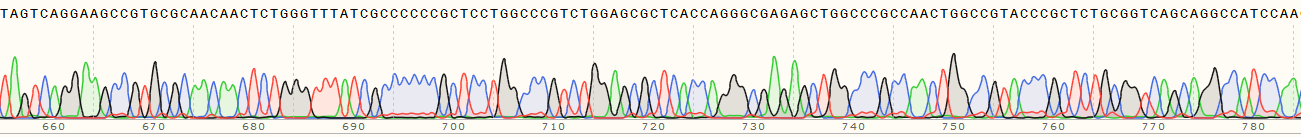


DH5a-JN-M-seq4871-Premix-primer_B12：


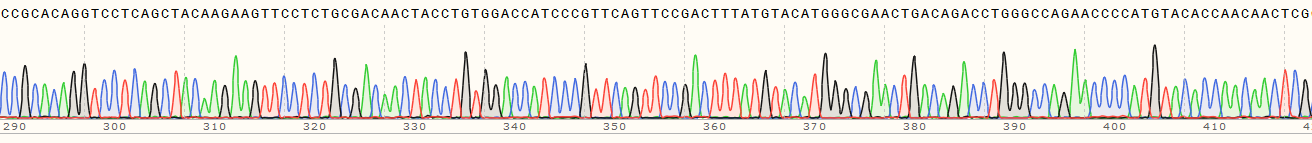


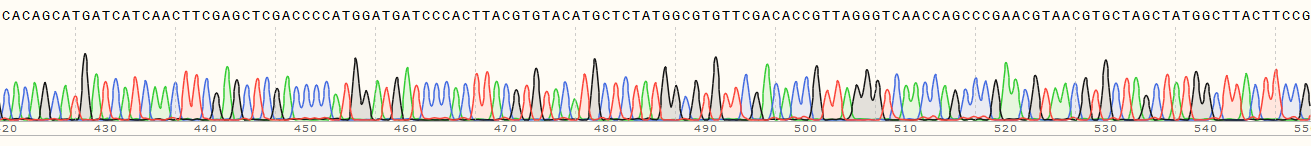


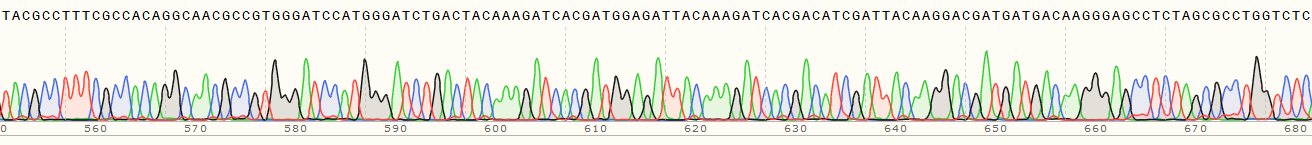


Original sequencing sequence of pLVX-Puro-MCS-3flag-TST(+)-hexon

CGTCAGATCGCCTGGAGACGCCATCCACGCTGTTTTGACCTCCATAGAAGACACCGACTCTACTAGAGGATCGCTAGCGCTACCGGACTCAGATCTCGAGATGGCGGCCCTCACGCCCGACCTGACTACCGCGACTCCGCGGCTCCAGTATTTTCACATCGCGGGCCCCGGGACGCGCGAATACCTCTCTGAGGACCTCCAACAGTTCATTTCCGCCACCGGAAGCTACTTTGACTTGAAAAACAAGTTCAGACAGACGGTCGTGGCGCCCACCCGAAATGTCACGACAGAAAAGGCTCAACGGCTGCAAATCCGCTTTTACCCCATCCAAACCGACGACACGTCGACGGGCTACCGCGTGCGGTACAACATCAATGTGGGCGACGGTTGGGTCCTGGACATGGGGTCGACCTATTTCGACATCAAGGGAATCCTAGACCGAGGGCCGTCCTTCAAGCCCTACTGCGGCACGGCTTACAACCCGCTGGCTCCCAAGGAGTCCATGTTTAACAACTGGTCGGAGACGGCACCCGGGCAGAACGTGTCCGCCTCCGGTCAGCTGTCCAACGTCTATACCAACACGAGCACCTCCAAAGACACGACGGCGGCGCAGGTGACGAAGATTTCCGGCGTCTTCCCCAATCCCAACCAGGGACCCGGAAGAAATCCTCTGCGACGGGTACAAAACGCCAACACCGGCGTGCTCGGTCGCTTCGCCAAGTCTCAGTACAATTACGCTTACGGTGCCTACGTCAAGCCCGTCGCCGCCGACGGTTCCCAGTCCCTCACGCAGACCCCCTACTGGATCATGGATAACACGGGCACCAATTACCTGGGAGCGGTGGCCGTCGAGGACTACACCAACAGCCTCTCGTACCCAGATACCATAGTCGTGCCGCCTCCCGAGGACTACGACGATTATAACATAGGCACCACGCGTGCGCTCAGGCCCAACTACATCGGGTTCAGGGATAACTTCATTAACCTGCTGTATCACGACTCCGGCGTGTGCTCGGGCACCCTCAACTCGGAGCGTTCGGGCATGAACGTGGTGGTCGAGCTGCCCGACCGGAATACCGAGCTCAGCTACCAGTACATGCTGGCCGACATGATGTCCCGCCATCACTATTTCGCCCTGTGGAACCAGGCCGTGGACCAGTACGACCCCGAGGTGCGAGTCTTCTCCAATGACGGTTACGAAGAAGGCGCGCCCAGCTACGCCTTCAACCCCGAAGCGGTAGGCGCGGGAGAAGGCTACGGCCCCGATCTCAGTCAGATTAAACTCTACACCAACAACACCGCCGCGAACGACAAAAACACCGCCGTGGCTAACGCCACTACCAACTTCTACTTCGGCACGGTACCCTCCTACGAAATCGATATCAGCGCTACCCAGAGGCGCAACTTTATCATGGCCAACATCGCCGAGTATCTGCCCGACCGTTACAAGTTTAGCATCTCCGGCTTCGACGCCACCAGCGTCGCGCCTACCACCTACGAGTACATGAACAAGCGCGTCCCCCTCACCAACGTCGTCGACATGTTCACGAACGTGGGTGCGCGTTGGTCCATCGACCAGATGGACAACGTCAACCCCTTCAACCACCACAGAAACTGGGGGCTGAAATACCGCTCCCAGCTGCTGGGAAACAGTCGCTACGTCAACTTCCACATCCAAGTGCCCCAAAAATTCTTCGCCATCAAAAACCTGCTGCTGCTCTCCGGCTCGTACACCTACGAGTGGGTGCTGCGCAAAGACCCCAACATGATCCTCCAATCCAGTCTGGGCAATGACCTGCGCGCCGACGGCGCCAGCATCATCTACAACGAGGTGAACCTCATGGCCAACTTCATGCCCATGGATCACAACACCAGTAACCAGCTCGAGCTGATGCTGAGAAACGCCACCAACGATCAGACCTTCGTGGACTACCTGGGAGCCAAAAACGCTCTATACTCGGTGCCCGCGGGCTCCACCGCCCTCACCATCAACATTCCCGCTCGCACCTGGGAGGGGATGCGCGGGTGGTCCTTCACTCGCATCAAGGCGGCCGAGACGCCTCAGCTGGGCGCCCAGTACGACGTCAACTTCAAGTACTCGGGCAGCATCGCCTACTCAGATGGAGGCTTCTACCTCTCGCACACCTTCCGTAACATGAGCATCCTCTTCGACACGTCCATCAACTGGCCGGGCAACGACCGGTTGCTCACGCCTAACATGTTCGAGATCAAGCGCTCGGTGGCGCTCGACACCGAGGGCTTCACCATGAGCCAGTGCGACATCACCAAGGACTGGTACCTGATCCAGATGGCCACGAACTACAACTTCGTCTATAACGGCTATCGATTCTGGCCCGATCGTCAGTACTTCCACTACGACTTCCTGCGAAATTTCGACCCCATGACGCGCCAGGGACCCAACTTCGCATTGCCCGGCCTCTTCGACCTCGTGTCTTACACCCCTACCACGGACAACAGCGGACAGCAGCCTAGTCAGGAAGCCGTGCGCAACAACTCTGGGTTTATCGCCCCCCGCTCCTGGCCCGTCTGGAGCGCTCACCAGGGCGAGAGCTGGCCCGCCAACTGGCCGTACCCGCTCTGCGGTCAGCAGGCCATCCAACCCGCACAGGTCCTCAGCTACAAGAAGTTCCTCTGCGACAACTACCTGTGGACCATCCCGTTCAGTTCCGACTTTATGTACATGGGCGAACTGACAGACCTGGGCCAGAACCCCATGTACACCAACAACTCGCACAGCATGATCATCAACTTCGAGCTCGACCCCATGGATGATCCCACTTACGTGTACATGCTCTATGGCGTGTTCGACACCGTTAGGGTCAACCAGCCCGAACGTAACGTGCTAGCTATGGCTTACTTCCGTACGCCTTTCGCCACAGGCAACGCCGTGGGATCCATGGGATCTGACTACAAAGATCACGATGGAGATTACAAAGATCACGACATCGATTACAAGGACGATGATGACAAGGGAGCCTCTAGCGCCTGGTCTC

According to the clone sequencing results, the sequencing results (highlighted part) matched the expected sequence 100%.

Original sequencing peak mapping of pLVX-Puro-3Flag-N-EGFP-CCT5-EGFP

CCT5-JC-R：

pEGFP-N5：
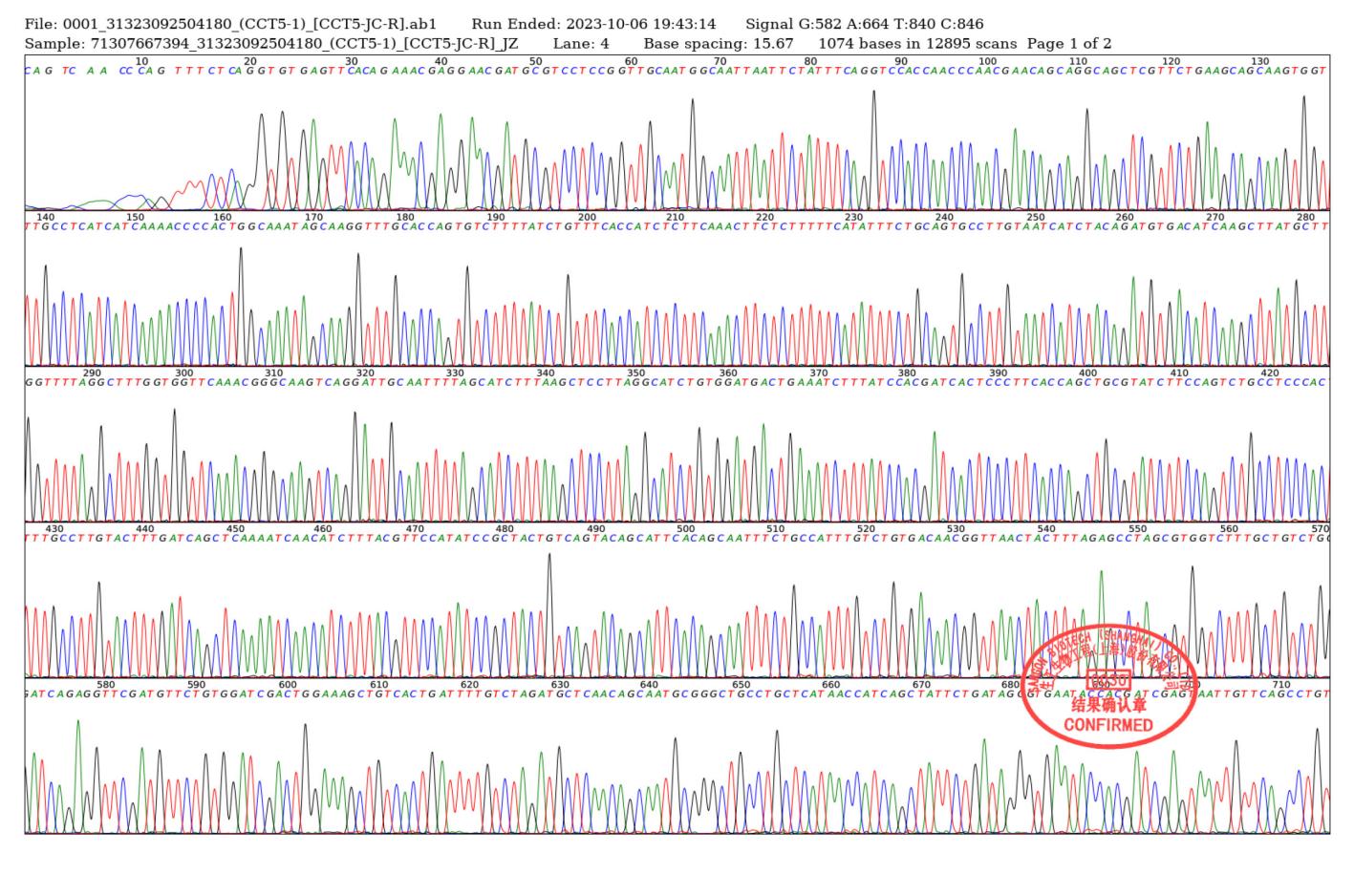


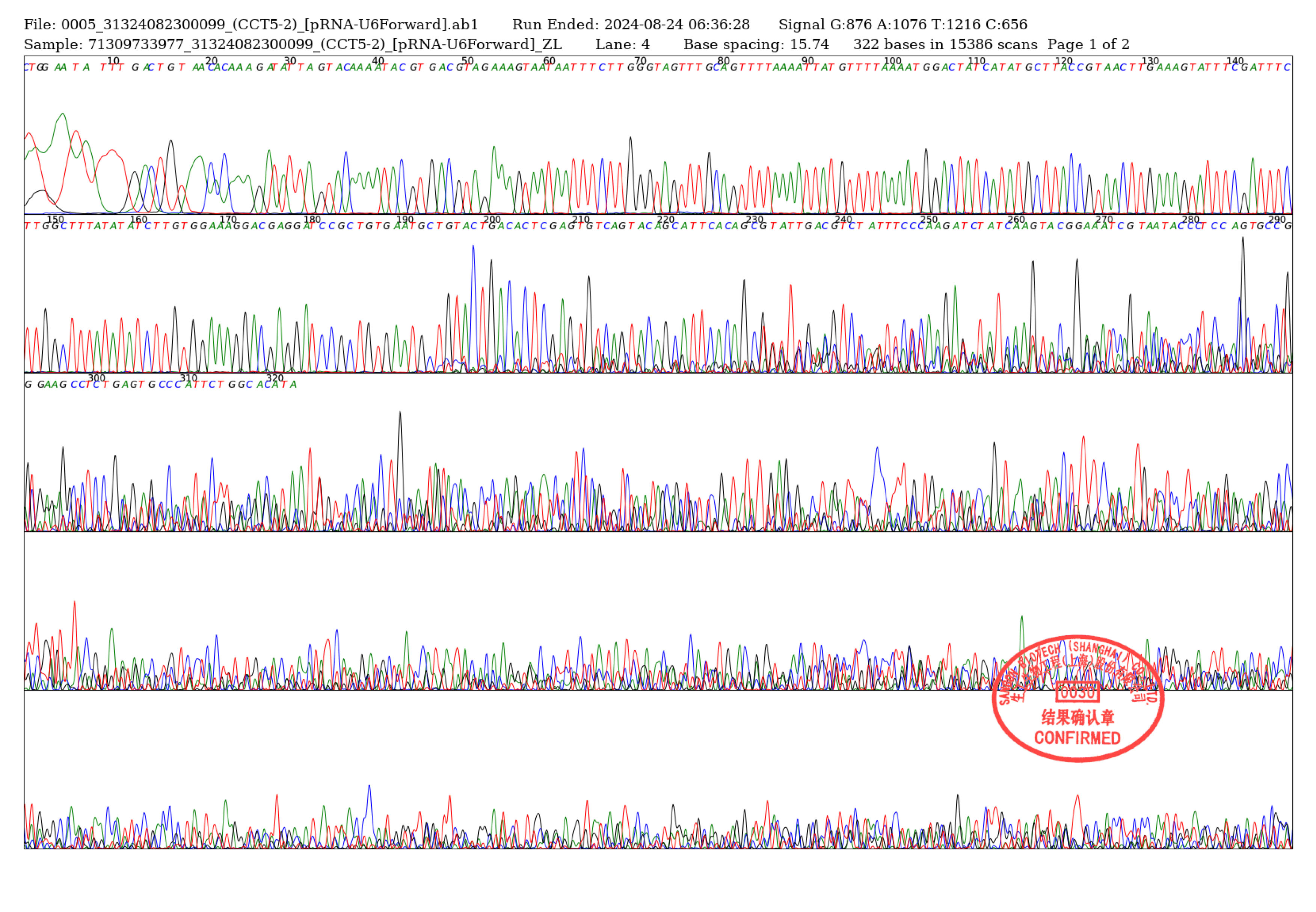


PMSCV-R：


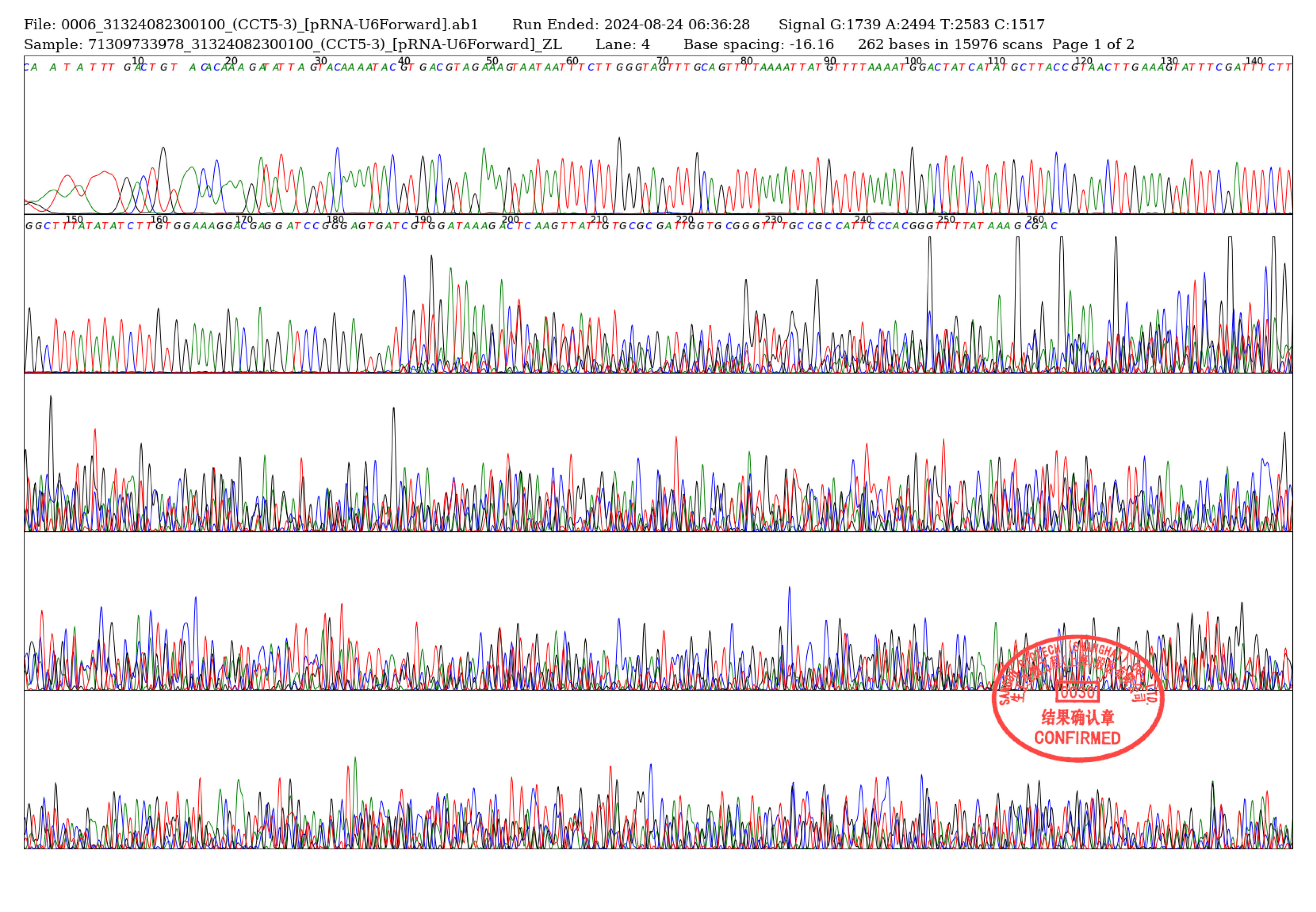


Original sequencing sequence of pLVX-Puro-3Flag-N-EGFP-CCT5-EGFP

TTTTGACCTCCATAGAAGACACCGACTCTACTAGAGGATCGCTAGCGCTACCGGACTCAGATCTCGAGATGGTGAGCAAGGGCGAGGAGCTGTTCACCGGGGTGGTGCCCATCCTGGTCGAGCTGGACGGCGACGTAAACGGCCACAAGTTCAGCGTGTCCGGCGAGGGCGAGGGCGATGCCACCTACGGCAAGCTGACCCTGAAGTTCATCTGCACCACCGGCAAGCTGCCCGTGCCCTGGCCCACCCTCGTGACCACCCTGACCTACGGCGTGCAGTGCTTCAGCCGCTACCCCGACCACATGAAGCAGCACGACTTCTTCAAGTCCGCCATGCCCGAAGGCTACGTCCAGGAGCGCACCATCTTCTTCAAGGACGACGGCAACTACAAGACCCGCGCCGAGGTGAAGTTCGAGGGCGACACCCTGGTGAACCGCATCGAGCTGAAGGGCATCGACTTCAAGGAGGACGGCAACATCCTGGGGCACAAGCTGGAGTACAACTACAACAGCCACAACGTCTATATCATGGCCGACAAGCAGAAGAACGGCATCAAGGTGAACTTCAAGATCCGCCACAACATCGAGGACGGCAGCGTGCAGCTCGCCGACCACTACCAGCAGAACACCCCCATCGGCGACGGCCCCGTGCTGCTGCCCGACAACCACTACCTGAGCACCCAGTCCGCCCTGAGCAAAGACCCCAACGAGAAGCGCGATCACATGGTCCTGCTGGAGTTCGTGACCGCCGCCGGGATCACTCTCGGCATGGACGAGCTGTACAAGGGCGGTTCATCGGCCATGGGGACGCTGGCGTTTGATGAGTATGGGCGGCCCTTTCTCATCCTCAAGGACCAGGAGCGCAAGACGCGTCTCATGGGGCTCGAGGCTCTCAAGTCTCACATCATGGCAGCAAAGGCTGTGGCAAGTACTCTGAGAACATCCCTTGGGCCCAATGGCTTGGATAAGATGATGGTGGACAAAGATGGTGAGGTGACAGTGACAAACGACGGAGCCACCATCCTGAACATGATGGACGTGGATCATCAGATAGCCAAGCTTATGGTGGAGCTGGCCAAATCTCAGGATGATGAGATTGGGGATGGAACCACTGGAGTCGTGGTTCTGGCTGGAGCATTATTGGAACAGGCTGAACAATTACTCGATCGTGGTATTCACCCTATCAGAATAGCTGATGGTTATGAGCAGGCAGCCCGCATTGCTGTTGAGCATCTAGACAAAATCAGTGACAGCTTTCCAGTCGATCCACAGAACATCGAACCTCTGATCCAGACAGCAAAGACCACGCTAGGCTCTAAAGTAGTTAACCGTTGTCACAGACAAATGGCAGAAATTGCTGTGAATGCTGTACTGACAGTAGCGGATATGGAACGTAAAGATGTTGATTTTGAGCTGATCAAAGTACAAGGCAAAGTGGGAGGCAGACTGGAAGATACGCAGCTGGTGAAGGGAGTGATCGTGGATAAAGATTTCAGTCATCCACAGATGCCTAAGGAGCTTAAAGATGCTAAAATTGCAATCCTGACTTGCCCGTTTGAACCACCAAAGCCTAAAACCAAGCATAAGCTTGATGTCACATCTGTAGATGATTACAAGGCACTGCAGAAATATGAAAAAGAGAAGTTTGAAGAGATGGTGAAACAGATAAAAGACACTGGTGCAAACCTTGCTATTTGCCAGTGGGGTTTTGATGATGAGGCAAACCACTTGCTGCTTCAGAACGAGCTGCCTGCTGTTCGTTGGGTTGGTGGACCTGAAATAGAATTAATTGCCATTGCAACCGGAGGACGCATCGTTCCTCGTTTCTGTGAACTCACACCTGAGAAACTGGGTTTTGCTGGTATTGTCAGAGAGATCTCCTTTGGCACAACGAAGGACAGAATGCTTGTCATTGAACAGTGCCAGAATTCTAGAGCTGTGACCATTTTCATCAGAGGAGGAAATAAAATGATAATTGAAGAAGCAAAGCGATCTCTCCATGATGCGCTGTGTGTGATCCGGAATCTCGTTCGCGATAACCGCATTGTGTACGGTGGTGGTGCAGCTGAAATTTCTTGTGCCTTGGCAGTCAGTGAAGCAGCAGATAAGTGCCCATCTTTGGAACAGTATGCAATGAGGGCGTTTGCAGATGCCCTGGAGGTAATCCCCATGGCCCTTTCGGAGAACAGTGGTATGAATCCAATTCAGACAATGACTGAAGTACGGGCGAGGCAAGTGAAGGAAAACAATCCCGCTCTTGGCATTGATTGTTTGCAGAAAGGAACAAACGATATGAAACAGCAGCATGTTATAGAAACCTTGATTGGTAAGAAACAACAGATTTCTCTGGCAACTCAGGTCGTTAGGATGATTCTGAAGATTGATGATATCCGTAGGCCTGGGAGAATCTGAAGAGGATCCGACTACAAAGACCATGACGGTGATTATAAAGATCATGACATCGATTACAAGGATGACGATGACAAGTGATCTAGATAATTCTACCGGGTAGGGGAGGCGCTTTTCCCAAGGCAGTCTGGAGCATGCGCTTTAGCAGCCCCGCTGGGCACTTGGCGCTACACAAGTGGCCTCTGGCCTCGCACACATTCCACATCCACCGGTAGGCGCCAACC

According to the clone sequencing results, the sequencing results (highlighted part) matched the expected sequence 100%.

Original sequencing peak mapping of pLVX-shRNA2-Puro-CCT5

(CCT5-1)_[pRNA-U6Forward]_H


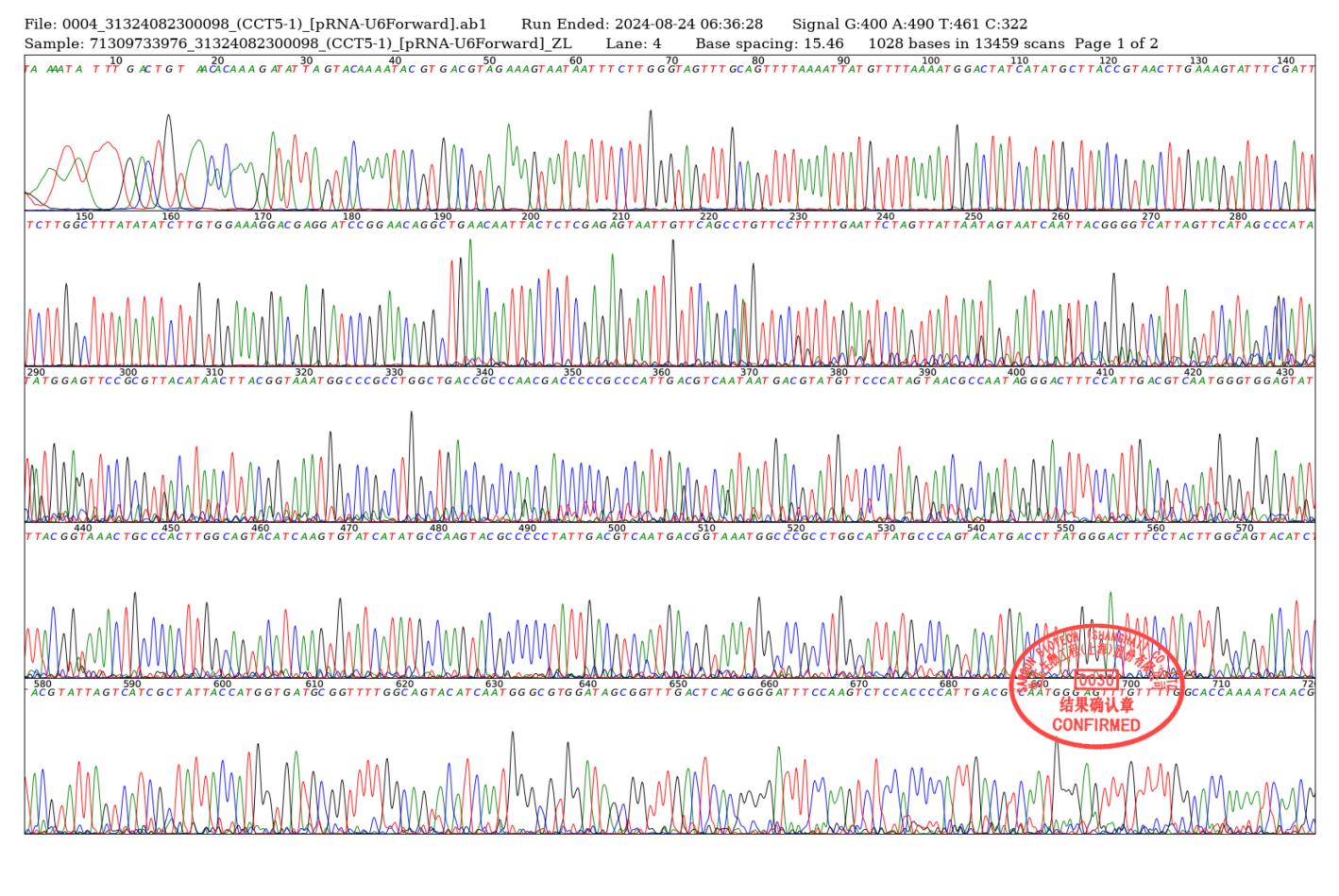
(CCT5-2)_[pRNA-U6Forward]_H


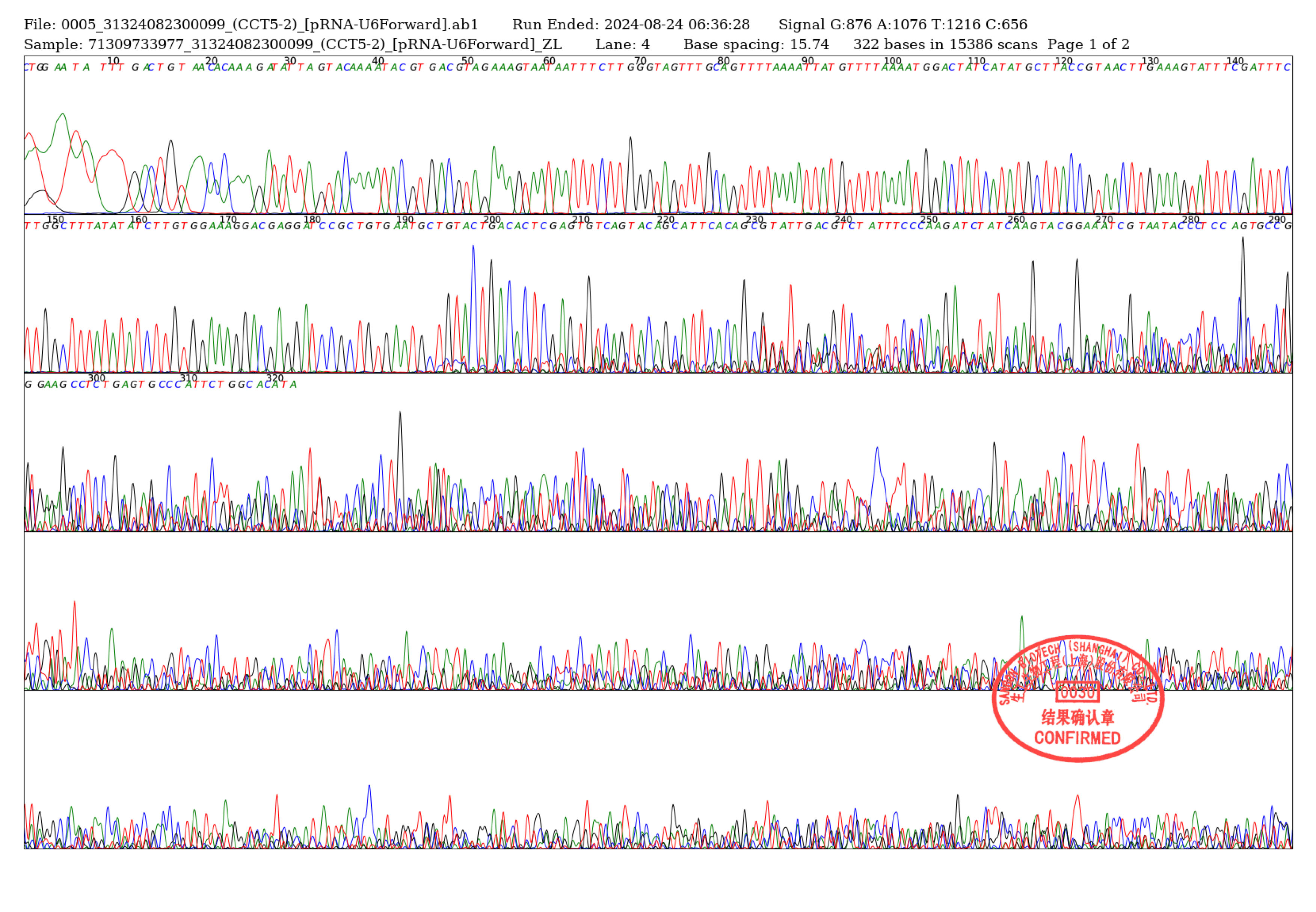


(CCT5-3)_[pRNA-U6Forward]


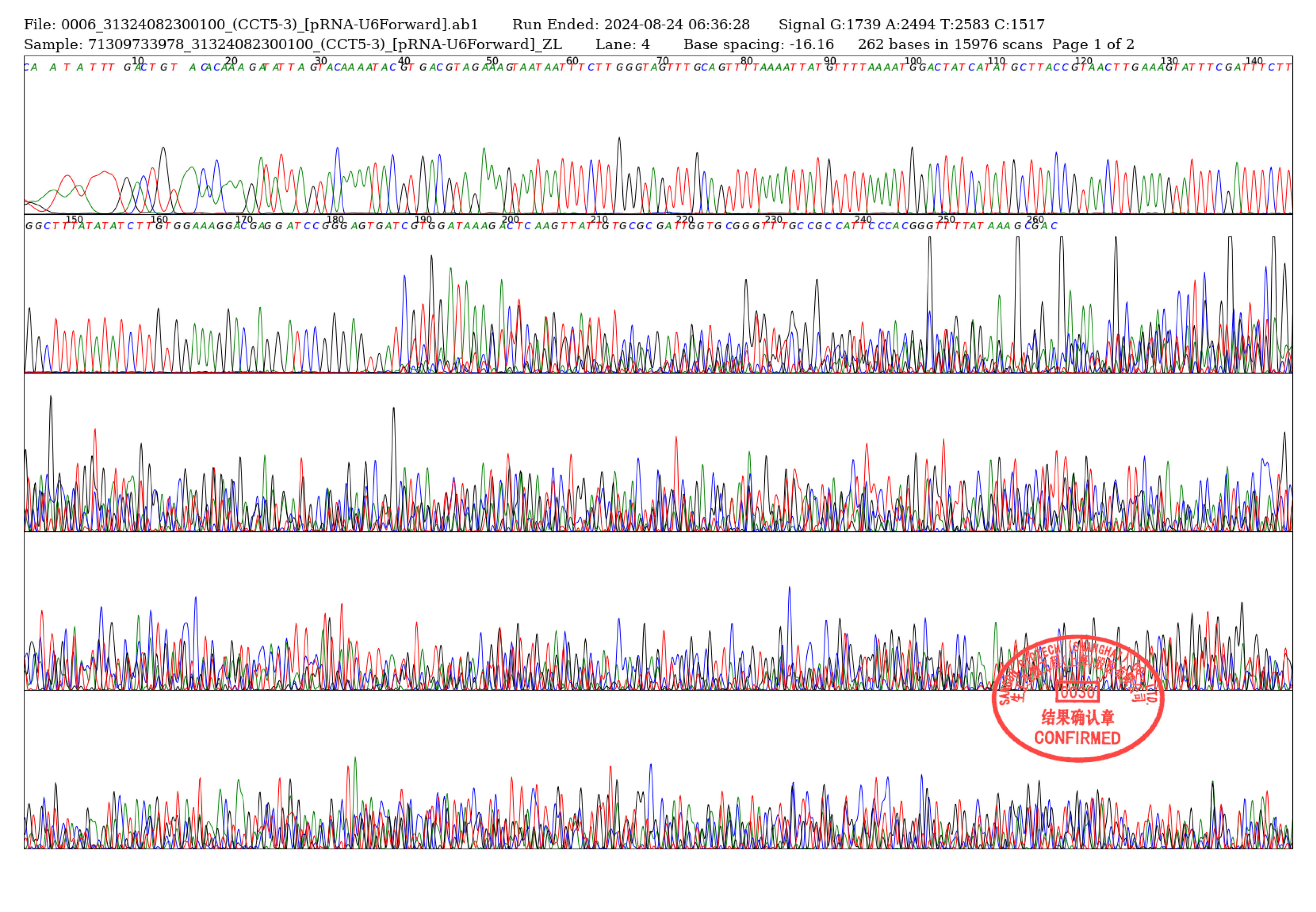

Supplement: Supplementary file 1 [file Supplementary_file_1.docx]
