# Supplementary material for: Unveiling the role of hexon-associated host proteins in fowl adenovirus serotype 4 replication
Source: Front Vet Sci. 2025 Jun 3;12:1562872. doi: 10.3389/fvets.2025.1562872 (PMC12170584; doi:10.3389/fvets.2025.1562872)
Supplement: Supplementary file 2 [file Supplementary_file_2.docx]

Supplementary file S2

1-82 proteins interacting with the hexon were screened

| **%Cov** | **%Cov(50)** | **%Cov(95)** | **Accession** | **Citable Accession** | **Gene name** | **Name** | **Species** | **Peptides(95%)** |
| --- | --- | --- | --- | --- | --- | --- | --- | --- |
| 34.00999904 | 26.01999938 | 19.14000064 | tr\|A0A1L1RY35\|A0A1L1RY35_CHICK |  | ATP5A1Z | ATP synthase subunit alpha OS=Gallus gallus OX=9031 GN=ATP5A1Z PE=3 SV=2 | CHICK | 9 |
| 8.85400027 | 8.85400027 | 5.208000168 | tr\|A0A1D5NVI1\|A0A1D5NVI1_CHICK | A0A1D5NVI1 | RPL9 | 60S ribosomal protein L9 OS=Gallus gallus OX=9031 GN=RPL9 PE=3 SV=1 | CHICK | 1 |
| 7.337000221 | 4.348000139 | 4.348000139 | tr\|A0A1D5NXB3\|A0A1D5NXB3_CHICK | A0A1D5NXB3 | LOC100859276 | Uncharacterized protein OS=Gallus gallus OX=9031 GN=LOC100859276 PE=4 SV=2 | CHICK | 1 |
| 39.05999959 | 39.05999959 | 35.49000025 | tr\|A0A1D5NXV1\|A0A1D5NXV1_CHICK | A0A1D5NXV1 | LOC100857858 | Tubulin alpha chain OS=Gallus gallus OX=9031 GN=LOC100857858 PE=3 SV=2 | CHICK | 25 |
| 14.2900005 | 14.2900005 | 14.2900005 | tr\|A0A1D5NZ06\|A0A1D5NZ06_CHICK | A0A1D5NZ06 | RPS27 | 40S ribosomal protein S27 OS=Gallus gallus OX=9031 GN=RPS27 PE=3 SV=1 | CHICK | 1 |
| 37.54999936 | 37.54999936 | 34.11999941 | tr\|A0A1D5P198\|A0A1D5P198_CHICK | A0A1D5P198 | LOC100859737 | Tubulin alpha chain OS=Gallus gallus OX=9031 GN=LOC100859737 PE=3 SV=2 | CHICK | 24 |
| 6.914000213 | 5.228000134 | 5.228000134 | tr\|A0A1D5P2Q1\|A0A1D5P2Q1_CHICK | A0A1D5P2Q1 | SACM1L | SAC domain-containing protein OS=Gallus gallus OX=9031 GN=SACM1L PE=4 SV=2 | CHICK | 2 |
| 21.34999931 | 12.91999966 | 12.91999966 | tr\|A0A1D5P3B1\|A0A1D5P3B1_CHICK | A0A1D5P3B1 | RPL11 | 60S ribosomal protein L11 OS=Gallus gallus OX=9031 GN=RPL11 PE=3 SV=1 | CHICK | 2 |
| 5.860000104 | 3.024999984 | 3.024999984 | tr\|A0A1D5P592\|A0A1D5P592_CHICK | A0A1D5P592 | PCCB | Uncharacterized protein OS=Gallus gallus OX=9031 GN=PCCB PE=4 SV=1 | CHICK | 1 |
| 10.40999964 | 2.040999942 | 2.040999942 | tr\|A0A1D5P6Q8\|A0A1D5P6Q8_CHICK | A0A1D5P6Q8 | SLC2A1 | Solute carrier family 2, facilitated glucose transporter member 1 OS=Gallus gallus OX=9031 GN=SLC2A1 PE=3 SV=1 | CHICK | 1 |
| 6.25 | 2.082999982 | 2.082999982 | tr\|A0A1D5P709\|A0A1D5P709_CHICK | A0A1D5P709 | DNAJC7 | J domain-containing protein OS=Gallus gallus OX=9031 GN=DNAJC7 PE=4 SV=2 | CHICK | 1 |
| 30.28999865 | 30.28999865 | 30.28999865 | tr\|A0A1D5PAR5\|A0A1D5PAR5_CHICK | A0A1D5PAR5 | TUBA3E | Tubulin alpha chain OS=Gallus gallus OX=9031 GN=TUBA3E PE=3 SV=1 | CHICK | 16 |
| 19.96999979 | 15.11999965 | 12.0099999 | tr\|A0A1D5PBX7\|A0A1D5PBX7_CHICK | A0A1D5PBX7 | ACACA | Acetyl-CoA carboxylase OS=Gallus gallus OX=9031 GN=ACACA PE=4 SV=2 | CHICK | 25 |
| 5.006000027 | 2.807999961 | 2.807999961 | tr\|A0A1D5PFL9\|A0A1D5PFL9_CHICK | A0A1D5PFL9 | NUP93 | Nuclear pore complex protein Nup93 OS=Gallus gallus OX=9031 GN=NUP93 PE=3 SV=1 | CHICK | 2 |
| 28.56999934 | 16.39000028 | 12.64999956 | tr\|A0A1D5PHS2\|A0A1D5PHS2_CHICK | A0A1D5PHS2 | KRT18 | IF rod domain-containing protein OS=Gallus gallus OX=9031 GN=KRT18 PE=3 SV=2 | CHICK | 5 |
| 3.387000039 | 1.439000014 | 1.057999954 | tr\|A0A1D5PJY1\|A0A1D5PJY1_CHICK | A0A1D5PJY1 | SPTBN1 | Spectrin beta chain OS=Gallus gallus OX=9031 GN=SPTBN1 PE=3 SV=1 | CHICK | 2 |
| 18.75 | 7.895000279 | 5.262999982 | tr\|A0A1D5PNK3\|A0A1D5PNK3_CHICK | A0A1D5PNK3 | SLC25A11 | Uncharacterized protein OS=Gallus gallus OX=9031 GN=SLC25A11 PE=3 SV=2 | CHICK | 1 |
| 6.32700026 | 2.459999919 | 2.459999919 | tr\|A0A1D5PQU1\|A0A1D5PQU1_CHICK | A0A1D5PQU1 | FARSA | Phenylalanine--tRNA ligase OS=Gallus gallus OX=9031 GN=FARSA PE=3 SV=2 | CHICK | 1 |
| 9.206999838 | 6.061000004 | 5.245000124 | tr\|A0A1D5PS29\|A0A1D5PS29_CHICK | A0A1D5PS29 | EEF2 | Elongation factor 2 OS=Gallus gallus OX=9031 GN=EEF2 PE=3 SV=1 | CHICK | 3 |
| 3.023999929 | 3.023999929 | 3.023999929 | tr\|A0A1D5PTH2\|A0A1D5PTH2_CHICK | A0A1D5PTH2 | CYP20A1 | Uncharacterized protein OS=Gallus gallus OX=9031 GN=CYP20A1 PE=3 SV=2 | CHICK | 1 |
| 10.48000008 | 7.639999688 | 7.639999688 | tr\|A0A1D5PU80\|A0A1D5PU80_CHICK | A0A1D5PU80 | ATP1A1 | Sodium/potassium-transporting ATPase subunit alpha OS=Gallus gallus OX=9031 GN=ATP1A1 PE=3 SV=1 | CHICK | 5 |
| 17.38000065 | 5.959999934 | 4.139000177 | tr\|A0A1D5PVA4\|A0A1D5PVA4_CHICK | A0A1D5PVA4 | ATAD3B | AAA domain-containing protein OS=Gallus gallus OX=9031 GN=ATAD3B PE=4 SV=1 | CHICK | 2 |
| 15.37999958 | 8.173000067 | 8.173000067 | tr\|A0A1D5PVL4\|A0A1D5PVL4_CHICK | A0A1D5PVL4 | EIF3F | MPN domain-containing protein OS=Gallus gallus OX=9031 GN=EIF3F PE=4 SV=2 | CHICK | 1 |
| 6.627000123 | 3.613999858 | 3.613999858 | tr\|A0A1D5PWW3\|A0A1D5PWW3_CHICK | A0A1D5PWW3 | HSD17B7 | Uncharacterized protein OS=Gallus gallus OX=9031 GN=HSD17B7 PE=4 SV=1 | CHICK | 1 |
| 9.031999856 | 3.871000186 | 3.871000186 | tr\|A0A1D5PX68\|A0A1D5PX68_CHICK | A0A1D5PX68 | SLC25A1 | Uncharacterized protein OS=Gallus gallus OX=9031 GN=SLC25A1 PE=3 SV=1 | CHICK | 1 |
| 2.721999958 | 2.721999958 | 2.721999958 | tr\|A0A1D5PXI1\|A0A1D5PXI1_CHICK | A0A1D5PXI1 | ALDH4A1 | Multifunctional fusion protein OS=Gallus gallus OX=9031 GN=ALDH4A1 PE=3 SV=1 | CHICK | 1 |
| 44.87000108 | 41.99000001 | 37.65999973 | tr\|A0A1D5PYK0\|A0A1D5PYK0_CHICK | A0A1D5PYK0 | HSPA8 | ，kOS=Gallus gallus OX=9031 GN=HSPA8 PE=3 SV=2 | CHICK | 53 |
| 8.608999848 | 4.636000097 | 4.636000097 | tr\|A0A1D5PZ84\|A0A1D5PZ84_CHICK | A0A1D5PZ84 | DHRS3 | Uncharacterized protein OS=Gallus gallus OX=9031 GN=DHRS3 PE=4 SV=2 | CHICK | 1 |
| 3.085000068 | 1.633000001 | 1.633000001 | tr\|A0A1D6UPR6\|A0A1D6UPR6_CHICK | A0A1D6UPR6 | SOAT1 | O-acyltransferase OS=Gallus gallus OX=9031 GN=SOAT1 PE=2 SV=1 | CHICK | 1 |
| 5.869000033 | 3.159999847 | 3.159999847 | tr\|A0A1I7Q412\|A0A1I7Q412_CHICK | A0A1I7Q412 | ENTPD5 | Ectonucleoside triphosphate diphosphohydrolase 5 OS=Gallus gallus OX=9031 GN=ENTPD5 PE=3 SV=1 | CHICK | 1 |
| 12.75999993 | 10.08000001 | 9.308999777 | tr\|A0A1L1RKB7\|A0A1L1RKB7_CHICK | A0A1L1RKB7 | ATP2A2 | Calcium-transporting ATPase OS=Gallus gallus OX=9031 GN=ATP2A2 PE=3 SV=2 | CHICK | 9 |
| 34.65999961 | 32.92999864 | 27.03999877 | tr\|A0A1L1RMM0\|A0A1L1RMM0_CHICK | A0A1L1RMM0 | TCP1 | CCT-alpha OS=Gallus gallus OX=9031 GN=TCP1 PE=3 SV=2 | CHICK | 16 |
| 8.948999643 | 4.540000111 | 4.540000111 | tr\|A0A1L1RND0\|A0A1L1RND0_CHICK | A0A1L1RND0 | HSP90AA1 | Heat shock protein HSP 90-alpha OS=Gallus gallus OX=9031 GN=HSP90AA1 PE=3 SV=2 | CHICK | 4 |
| 10.28999984 | 9.034000337 | 2.759999968 | tr\|A0A1L1RR71\|A0A1L1RR71_CHICK | A0A1L1RR71 | ALDH18A1 | Delta-1-pyrroline-5-carboxylate synthase OS=Gallus gallus OX=9031 GN=ALDH18A1 PE=3 SV=1 | CHICK | 2 |
| 19.4600001 | 14.85999972 | 14.85999972 | tr\|A0A1L1RUX2\|A0A1L1RUX2_CHICK | A0A1L1RUX2 | EIF4A2 | RNA helicase OS=Gallus gallus OX=9031 GN=EIF4A2 PE=3 SV=2 | CHICK | 5 |
| 14.1900003 | 8.139999956 | 6.744000316 | tr\|A0A1L1RXE9\|A0A1L1RXE9_CHICK | A0A1L1RXE9 | UQCRC2 | Uncharacterized protein OS=Gallus gallus OX=9031 GN=UQCRC2 PE=4 SV=2 | CHICK | 2 |
| 26.49999857 | 25.11999905 | 23.72999936 | tr\|A0A1L1RY04\|A0A1L1RY04_CHICK | A0A1L1RY04 | ATP5B | ATP synthase subunit beta OS=Gallus gallus OX=9031 GN=ATP5B PE=3 SV=2 | CHICK | 14 |
| 33.7199986 | 32.10000098 | 27.25000083 | tr\|A0A1X9WEL5\|A0A1X9WEL5_CHICK | A0A1X9WEL5 | TUFM | Elongation factor Tu OS=Gallus gallus OX=9031 GN=TUFM PE=2 SV=1 | CHICK | 14 |
| 5.485999957 | 1.496000029 | 1.496000029 | tr\|A0A3Q2TSF2\|A0A3Q2TSF2_CHICK | A0A3Q2TSF2 | -- | Uncharacterized protein OS=Gallus gallus OX=9031 PE=4 SV=1 | CHICK | 1 |
| 5.124000087 | 2.864000015 | 2.864000015 | tr\|A0A3Q2U150\|A0A3Q2U150_CHICK | A0A3Q2U150 | -- | Uncharacterized protein OS=Gallus gallus OX=9031 PE=4 SV=1 | CHICK | 3 |
| 8.190000057 | 4.309999943 | 4.309999943 | tr\|A0A3Q2U278\|A0A3Q2U278_CHICK | A0A3Q2U278 | SMC1A | Uncharacterized protein OS=Gallus gallus OX=9031 GN=SMC1A PE=4 SV=1 | CHICK | 1 |
| 13.16000074 | 7.895000279 | 7.895000279 | tr\|A0A3Q2U3Q5\|A0A3Q2U3Q5_CHICK | A0A3Q2U3Q5 | MAT2A | Methionine adenosyltransferase OS=Gallus gallus OX=9031 GN=MAT2A PE=3 SV=1 | CHICK | 1 |
| 7.374999672 | 2.60300003 | 2.60300003 | tr\|A0A3Q2U7I7\|A0A3Q2U7I7_CHICK | A0A3Q2U7I7 | LOC107051177 | RuvB-like helicase OS=Gallus gallus OX=9031 GN=LOC107051177 PE=3 SV=1 | CHICK | 1 |
| 20.8100006 | 16.60999954 | 12.73999959 | tr\|A0A3Q2UA96\|A0A3Q2UA96_CHICK | A0A3Q2UA96 | CCT3 | T-complex protein 1 subunit gamma OS=Gallus gallus OX=9031 GN=CCT3 PE=3 SV=1 | CHICK | 6 |
| 12.3999998 | 5.660000071 | 4.941999912 | tr\|A0A3Q2UDT6\|A0A3Q2UDT6_CHICK | A0A3Q2UDT6 | NPEPPS | Uncharacterized protein OS=Gallus gallus OX=9031 GN=NPEPPS PE=3 SV=1 | CHICK | 6 |
| 8.620999753 | 2.97299996 | 2.377999946 | tr\|A0A3Q2UKS1\|A0A3Q2UKS1_CHICK | A0A3Q2UKS1 | EPRS | Glutamyl-tRNA synthetase OS=Gallus gallus OX=9031 GN=EPRS PE=3 SV=1 | CHICK | 2 |
| 7.316999882 | 7.316999882 | 7.316999882 | tr\|A0A3Q3A3Q2\|A0A3Q3A3Q2_CHICK | A0A3Q3A3Q2 | -- | Uncharacterized protein OS=Gallus gallus OX=9031 PE=4 SV=1 | CHICK | 1 |
| 1.123000029 | 1.123000029 | 1.123000029 | tr\|A0A3Q3AQV2\|A0A3Q3AQV2_CHICK | A0A3Q3AQV2 | SEC31A | Protein transport protein Sec31A OS=Gallus gallus OX=9031 GN=SEC31A PE=3 SV=1 | CHICK | 1 |
| 9.75600034 | 9.75600034 | 9.75600034 | tr\|A0A3Q3AUG8\|A0A3Q3AUG8_CHICK | A0A3Q3AUG8 | LOC107050717 | Uncharacterized protein OS=Gallus gallus OX=9031 GN=LOC107050717 PE=3 SV=1 | CHICK | 1 |
| 6.556999683 | 1.405000035 | 1.405000035 | tr\|A0A3Q3AW40\|A0A3Q3AW40_CHICK | A0A3Q3AW40 | UNC45A | UNC45-central domain-containing protein OS=Gallus gallus OX=9031 GN=UNC45A PE=4 SV=1 | CHICK | 1 |
| 7.367999852 | 1.805000007 | 1.805000007 | tr\|A0A3Q3AXS8\|A0A3Q3AXS8_CHICK | A0A3Q3AXS8 | ILVBL | 2-hydroxyacyl-CoA lyase 2 OS=Gallus gallus OX=9031 GN=ILVBL PE=3 SV=1 | CHICK | 1 |
| 8.788999915 | 4.808999971 | 3.316999972 | tr\|A0A3S5ZPN3\|A0A3S5ZPN3_CHICK | A0A3S5ZPN3 | DDX5 | DEAD box protein 5 OS=Gallus gallus OX=9031 GN=DDX5 PE=3 SV=1 | CHICK | 2 |
| 14.49999958 | 14.49999958 | 14.49999958 | tr\|A0A452J7V2\|A0A452J7V2_CHICK | A0A452J7V2 | RAB2A | Ras-related protein Rab-2A OS=Gallus gallus OX=9031 GN=RAB2A PE=4 SV=1 | CHICK | 2 |
| 26.01999938 | 25.20000041 | 25.20000041 | tr\|A2NR64\|A2NR64_CHICK | A2NR64 | DAD1 | Dolichyl-diphosphooligosaccharide--protein glycosyltransferase subunit DAD1 OS=Gallus gallus OX=9031 GN=DAD1 PE=3 SV=1 | CHICK | 2 |
| 37.25000024 | 30.63000143 | 28.06999981 | AMQ13091.1 | AMQ13091.1 | -- | hexon protein [Fowl aviadenovirus 4] | Fowl aviadenovirus 4 | 44 |
| 11.30999997 | 11.30999997 | 11.30999997 | tr\|D2D3P4\|D2D3P4_CHICK | D2D3P4 | Rab27a | Small monomeric GTPase OS=Gallus gallus OX=9031 GN=Rab27a PE=2 SV=1 | CHICK | 2 |
| 7.568000257 | 7.568000257 | 7.568000257 | tr\|E1BQU4\|E1BQU4_CHICK | E1BQU4 | NSF | Vesicle-fusing ATPase OS=Gallus gallus OX=9031 GN=NSF PE=3 SV=2 | CHICK | 5 |
| 9.963999689 | 4.270000011 | 4.270000011 | tr\|E1BTV1\|E1BTV1_CHICK | E1BTV1 | STOM | PHB domain-containing protein OS=Gallus gallus OX=9031 GN=STOM PE=3 SV=3 | CHICK | 1 |
| 18.56999993 | 18.56999993 | 18.56999993 | tr\|E1BU66\|E1BU66_CHICK | E1BU66 | RPL38 | 60S ribosomal protein L38 OS=Gallus gallus OX=9031 GN=RPL38 PE=3 SV=3 | CHICK | 1 |
| 5.149000138 | 1.338999998 | 1.338999998 | tr\|E1BV44\|E1BV44_CHICK | E1BV44 | CSE1L | Chromosome segregation 1-like protein OS=Gallus gallus OX=9031 GN=CSE1L PE=3 SV=2 | CHICK | 1 |
| 17.13999957 | 17.13999957 | 10.71000025 | tr\|E1BY89\|E1BY89_CHICK | E1BY89 | RPL23 | 60S ribosomal protein L23 OS=Gallus gallus OX=9031 GN=RPL23 PE=3 SV=2 | CHICK | 2 |
| 2.269000001 | 0.33199999 | 0.33199999 | tr\|E1BYF5\|E1BYF5_CHICK | E1BYF5 | AFDN | Uncharacterized protein OS=Gallus gallus OX=9031 GN=AFDN PE=4 SV=4 | CHICK | 1 |
| 12.40999997 | 6.948000193 | 4.963000119 | tr\|E1BZ74\|E1BZ74_CHICK | E1BZ74 | AFG3L2 | AAA domain-containing protein OS=Gallus gallus OX=9031 GN=AFG3L2 PE=3 SV=3 | CHICK | 4 |
| 1.456999965 | 1.456999965 | 1.456999965 | tr\|E1C0C3\|E1C0C3_CHICK | E1C0C3 | SEC63 | J domain-containing protein OS=Gallus gallus OX=9031 GN=SEC63 PE=4 SV=2 | CHICK | 1 |
| 7.225000113 | 5.779999867 | 4.623999819 | tr\|E1C0F1\|E1C0F1_CHICK | E1C0F1 | RPN1 | Dolichyl-diphosphooligosaccharide--protein glycosyltransferase subunit 1 OS=Gallus gallus OX=9031 GN=RPN1 PE=3 SV=3 | CHICK | 3 |
| 22.80000001 | 9.73900035 | 9.73900035 | tr\|E1C0Q5\|E1C0Q5_CHICK | E1C0Q5 | ACAT1 | Uncharacterized protein OS=Gallus gallus OX=9031 GN=ACAT1 PE=3 SV=2 | CHICK | 3 |
| 3.830000013 | 0.84180003 | 0.84180003 | tr\|E1C1B8\|E1C1B8_CHICK | E1C1B8 | CNOT1 | Uncharacterized protein OS=Gallus gallus OX=9031 GN=CNOT1 PE=4 SV=2 | CHICK | 2 |
| 3.410999849 | 1.169999968 | 1.169999968 | tr\|E1C296\|E1C296_CHICK | E1C296 | LLGL2 | LLGL domain-containing protein OS=Gallus gallus OX=9031 GN=LLGL2 PE=3 SV=3 | CHICK | 1 |
| 7.458999753 | 3.866999969 | 3.866999969 | tr\|E1C3D2\|E1C3D2_CHICK | E1C3D2 | 2-Sep | Septin OS=Gallus gallus OX=9031 GN=SEPT2 PE=3 SV=3 | CHICK | 1 |
| 21.14000022 | 15.45999944 | 15.45999944 | tr\|E6N1V8\|E6N1V8_CHICK | E6N1V8 | GNB2L1 | Guanine nucleotide-binding protein beta subunit2-like 1 OS=Gallus gallus OX=9031 GN=GNB2L1 PE=4 SV=1 | CHICK | 4 |
| 5.449000001 | 2.563999966 | 2.563999966 | tr\|E6N1V9\|E6N1V9_CHICK | E6N1V9 | B-BTN1 | Similar to butyrophilin 1 and tripartite motif protein 39 OS=Gallus gallus OX=9031 GN=B-BTN1 PE=4 SV=1 | CHICK | 2 |
| 11.63000017 | 8.305999637 | 8.305999637 | tr\|F1N833\|F1N833_CHICK | F1N833 | PHB2 | Prohibitin OS=Gallus gallus OX=9031 GN=PHB2 PE=3 SV=4 | CHICK | 2 |
| 4.462999851 | 3.187999874 | 1.274999976 | tr\|F1N9U0\|F1N9U0_CHICK | F1N9U0 | PRPF6 | PRP6 homolog OS=Gallus gallus OX=9031 GN=PRPF6 PE=4 SV=4 | CHICK | 1 |
| 1.310999971 | 0.412000017 | 0.412000017 | tr\|F1NAK4\|F1NAK4_CHICK | F1NAK4 | GCN1 | TOG domain-containing protein OS=Gallus gallus OX=9031 GN=GCN1 PE=3 SV=4 | CHICK | 1 |
| 28.70999873 | 20.46000063 | 20.46000063 | tr\|F1NBD7\|F1NBD7_CHICK | F1NBD7 | CDK1 | Cyclin-dependent kinase 1 OS=Gallus gallus OX=9031 GN=CDK1 PE=3 SV=1 | CHICK | 5 |
| 2.864000015 | 2.864000015 | 2.864000015 | tr\|F1NBE3\|F1NBE3_CHICK | F1NBE3 | GCAT | Aminotran_1_2 domain-containing protein OS=Gallus gallus OX=9031 GN=GCAT PE=3 SV=2 | CHICK | 1 |
| 4.202000052 | 4.202000052 | 1.89100001 | tr\|F1NBN1\|F1NBN1_CHICK | F1NBN1 | SEC61A1 | Plug_translocon domain-containing protein OS=Gallus gallus OX=9031 GN=SEC61A1 PE=3 SV=2 | CHICK | 1 |
| 4.695000127 | 4.695000127 | 4.695000127 | tr\|F1NC27\|F1NC27_CHICK | F1NC27 | RAB21 | Ras-related protein Rab-21 OS=Gallus gallus OX=9031 GN=RAB21 PE=3 SV=3 | CHICK | 1 |
| 10.48000008 | 7.999999821 | 5.931000039 | tr\|F1NC33\|F1NC33_CHICK | F1NC33 | HSP90AB1 | Heat shock cognate protein HSP 90-beta OS=Gallus gallus OX=9031 GN=HSP90AB1 PE=3 SV=1 | CHICK | 4 |
| 7.28700012 | 1.923000067 | 1.923000067 | tr\|F1NCS6\|F1NCS6_CHICK | F1NCS6 | MTHFD1L | Formyltetrahydrofolate synthetase OS=Gallus gallus OX=9031 GN=MTHFD1L PE=3 SV=3 | CHICK | 1 |
| 8.374000341 | 5.17200008 | 4.18700017 | tr\|F1NFJ0\|F1NFJ0_CHICK | F1NFJ0 | MCM3 | DNA replication licensing factor MCM3 OS=Gallus gallus OX=9031 GN=MCM3 PE=3 SV=1 | CHICK | 3 |
| 8.488000184 | 3.548999876 | 3.548999876 | tr\|F1NGM2\|F1NGM2_CHICK | F1NGM2 | COQ8A | Atypical kinase COQ8A, mitochondrial OS=Gallus gallus OX=9031 GN=COQ8A PE=3 SV=2 | CHICK | 2 |
| 9.993000329 | 6.054000184 | 5.324999988 | tr\|F1NGU3\|F1NGU3_CHICK | F1NGU3 | LRPPRC | Uncharacterized protein OS=Gallus gallus OX=9031 GN=LRPPRC PE=4 SV=2 | CHICK | 6 |
| 39.50000107 | 25.20999908 | 25.20999908 | tr\|F1NH93\|F1NH93_CHICK | F1NH93 | RPS20 | 40S ribosomal protein S20 OS=Gallus gallus OX=9031 GN=RPS20 PE=3 SV=3 | CHICK | 4 |
| 3.007999994 | 1.950999908 | 1.950999908 | tr\|F1NHL2\|F1NHL2_CHICK | F1NHL2 | CAND1 | TIP120 domain-containing protein OS=Gallus gallus OX=9031 GN=CAND1 PE=3 SV=4 | CHICK | 2 |
| 21.96000069 | 9.347999841 | 6.304000318 | tr\|F1NJ08\|F1NJ08_CHICK | F1NJ08 | VIM | Vimentin OS=Gallus gallus OX=9031 GN=VIM PE=3 SV=1 | CHICK | 2 |
| 15.12999982 | 13.16000074 | 5.305000022 | tr\|F1NJC7\|F1NJC7_CHICK | F1NJC7 | ALDH1A1 | Retinal dehydrogenase 1 OS=Gallus gallus OX=9031 GN=ALDH1A1 PE=3 SV=2 | CHICK | 2 |
| 5.330999941 | 3.492999822 | 2.205999941 | tr\|F1NK38\|F1NK38_CHICK | F1NK38 | CCT7 | T-complex protein 1 subunit eta OS=Gallus gallus OX=9031 GN=CCT7 PE=3 SV=3 | CHICK | 1 |
| 8.632999659 | 8.632999659 | 8.632999659 | tr\|F1NLW3\|F1NLW3_CHICK | F1NLW3 | -- | Mitochondrial import receptor subunit TOM22 homolog OS=Gallus gallus OX=9031 PE=3 SV=3 | CHICK | 1 |
| 5.53399995 | 3.35999988 | 3.35999988 | tr\|F1NNE0\|F1NNE0_CHICK | F1NNE0 | DNAJA3 | Uncharacterized protein OS=Gallus gallus OX=9031 GN=DNAJA3 PE=3 SV=5 | CHICK | 1 |
| 36.62999868 | 32.10000098 | 20.99000067 | tr\|F1NPA9\|F1NPA9_CHICK | F1NPA9 | RPS3 | DNA-(apurinic or apyrimidinic site) lyase OS=Gallus gallus OX=9031 GN=RPS3 PE=3 SV=2 | CHICK | 4 |
| 3.609000146 | 1.654000022 | 1.654000022 | tr\|F1NPJ4\|F1NPJ4_CHICK | F1NPJ4 | SDHA | Succinate dehydrogenase [ubiquinone] flavoprotein subunit, mitochondrial OS=Gallus gallus OX=9031 GN=SDHA PE=3 SV=2 | CHICK | 1 |
| 7.27699995 | 2.58200001 | 2.58200001 | tr\|F1NRU5\|F1NRU5_CHICK | F1NRU5 | NOB1 | RNA-binding protein NOB1 OS=Gallus gallus OX=9031 GN=NOB1 PE=3 SV=2 | CHICK | 1 |
| 1.744000055 | 1.744000055 | 1.744000055 | tr\|F1NSY0\|F1NSY0_CHICK | F1NSY0 | RIOK3 | Serine/threonine-protein kinase RIO3 OS=Gallus gallus OX=9031 GN=RIOK3 PE=3 SV=4 | CHICK | 1 |
| 7.096999884 | 7.096999884 | 7.096999884 | tr\|F1NT14\|F1NT14_CHICK | F1NT14 | MGST1 | Glutathione transferase OS=Gallus gallus OX=9031 GN=MGST1 PE=3 SV=2 | CHICK | 1 |
| 1.546000037 | 1.546000037 | 1.546000037 | tr\|F1NTM6\|F1NTM6_CHICK | F1NTM6 | TFRC | Transferrin receptor protein 1 OS=Gallus gallus OX=9031 GN=TFRC PE=3 SV=4 | CHICK | 1 |
| 18.05000007 | 12.03000024 | 6.01500012 | tr\|F1NU06\|F1NU06_CHICK | F1NU06 | MPC2 | Mitochondrial pyruvate carrier OS=Gallus gallus OX=9031 GN=MPC2 PE=3 SV=2 | CHICK | 1 |
| 2.696999907 | 2.696999907 | 2.696999907 | tr\|F1NUY5\|F1NUY5_CHICK | F1NUY5 | FAF2 | UBX domain-containing protein OS=Gallus gallus OX=9031 GN=FAF2 PE=4 SV=2 | CHICK | 1 |
| 4.140000045 | 4.140000045 | 1.699000038 | tr\|F1NV33\|F1NV33_CHICK | F1NV33 | MSH2 | DNA mismatch repair protein OS=Gallus gallus OX=9031 GN=MSH2 PE=3 SV=3 | CHICK | 1 |
| 8.021000028 | 4.100000113 | 2.316999994 | tr\|F1NWF6\|F1NWF6_CHICK | F1NWF6 | ASNS | Asparagine synthetase [glutamine-hydrolyzing] OS=Gallus gallus OX=9031 GN=ASNS PE=4 SV=1 | CHICK | 1 |
| 47.18999863 | 42.25000143 | 36.84999943 | tr\|F1NYB1\|F1NYB1_CHICK | F1NYB1 | TUBB4B | Tubulin beta chain OS=Gallus gallus OX=9031 GN=TUBB4B PE=3 SV=4 | CHICK | 29 |
| 6.452000141 | 6.452000141 | 6.452000141 | tr\|F1NYM3\|F1NYM3_CHICK | F1NYM3 | NDUFS7 | Complex I-20kD OS=Gallus gallus OX=9031 GN=NDUFS7 PE=3 SV=3 | CHICK | 1 |
| 7.034999877 | 2.345000021 | 2.345000021 | tr\|F1NYW0\|F1NYW0_CHICK | F1NYW0 | PTPN2 | Uncharacterized protein OS=Gallus gallus OX=9031 GN=PTPN2 PE=4 SV=4 | CHICK | 1 |
| 20.89000046 | 14.37000036 | 14.37000036 | tr\|F1NZ86\|F1NZ86_CHICK | F1NZ86 | HSPA9 | 75 kDa glucose-regulated protein OS=Gallus gallus OX=9031 GN=HSPA9 PE=3 SV=1 | CHICK | 7 |
| 3.926999867 | 2.104000002 | 2.104000002 | tr\|F1P0M2\|F1P0M2_CHICK | F1P0M2 | PCCA | Propanoyl-CoA:carbon dioxide ligase subunit alpha OS=Gallus gallus OX=9031 GN=PCCA PE=4 SV=2 | CHICK | 1 |
| 43.36999953 | 39.77999985 | 31.00999892 | tr\|G1K338\|G1K338_CHICK | G1K338 | TUBB2A | Tubulin beta chain OS=Gallus gallus OX=9031 GN=TUBB2A PE=3 SV=3 | CHICK | 22 |
| 7.090000063 | 4.645000026 | 4.645000026 | tr\|H9L0M5\|H9L0M5_CHICK | H9L0M5 | DAP3 | Uncharacterized protein OS=Gallus gallus OX=9031 GN=DAP3 PE=3 SV=4 | CHICK | 1 |
| 52.34000087 | 45.30999959 | 33.59000087 | tr\|O42388\|O42388_CHICK | O42388 | UBA52 | 60S ribosomal protein L40 OS=Gallus gallus OX=9031 GN=UBA52 PE=2 SV=1 | CHICK | 6 |
| 4.898000136 | 3.128999844 | 1.224000007 | tr\|O42484\|O42484_CHICK | O42484 | HSD17B4 | 17-beta-hydroxysteroid dehydrogenase type IV OS=Gallus gallus OX=9031 GN=HSD17B4 PE=2 SV=1 | CHICK | 1 |
| 48.19999933 | 43.23999882 | 37.83999979 | sp\|P09244\|TBB7_CHICK | P09244 | -- | Tubulin beta-7 chain OS=Gallus gallus OX=9031 PE=2 SV=1 | CHICK | 30 |
| 30.27000129 | 24.66000021 | 19.95999962 | sp\|P09653\|TBB5_CHICK | P09653 | -- | Tubulin beta-5 chain OS=Gallus gallus OX=9031 PE=3 SV=1 | CHICK | 14 |
| 16.58000052 | 16.58000052 | 16.58000052 | sp\|P0CB50\|PRDX1_CHICK | P0CB50 | PRDX1 | Peroxiredoxin-1 OS=Gallus gallus OX=9031 GN=PRDX1 PE=1 SV=1 | CHICK | 3 |
| 14.50999975 | 14.50999975 | 10.45000032 | sp\|P21642\|PCKGM_CHICK | P21642 | PCK2 | Phosphoenolpyruvate carboxykinase [GTP], mitochondrial OS=Gallus gallus OX=9031 GN=PCK2 PE=1 SV=2 | CHICK | 6 |
| 44.26999986 | 40.66999853 | 31.90999925 | sp\|P32882\|TBB2_CHICK | P32882 | -- | Tubulin beta-2 chain OS=Gallus gallus OX=9031 PE=1 SV=1 | CHICK | 23 |
| 20.82999945 | 20.82999945 | 20.82999945 | sp\|P42558\|RAN_CHICK | P42558 | RAN | GTP-binding nuclear protein Ran OS=Gallus gallus OX=9031 GN=RAN PE=2 SV=1 | CHICK | 5 |
| 6.4790003 | 6.4790003 | 6.4790003 | sp\|P50147\|GNAI2_CHICK | P50147 | GNAI2 | Guanine nucleotide-binding protein G(i) subunit alpha-2 OS=Gallus gallus OX=9031 GN=GNAI2 PE=2 SV=2 | CHICK | 2 |
| 3.105000034 | 0.817000028 | 0.817000028 | tr\|Q5F354\|Q5F354_CHICK | Q5F354 | RCJMB04_34d13 | Coatomer subunit alpha OS=Gallus gallus OX=9031 GN=RCJMB04_34d13 PE=2 SV=1 | CHICK | 1 |
| 2.988000028 | 1.992000081 | 1.992000081 | tr\|Q5F3G9\|Q5F3G9_CHICK | Q5F3G9 | RCJMB04_17g4 | Dihydrolipoamide acetyltransferase component of pyruvate dehydrogenase complex OS=Gallus gallus OX=9031 GN=RCJMB04_17g4 PE=2 SV=1 | CHICK | 1 |
| 10.09000018 | 10.09000018 | 10.09000018 | tr\|Q5F3R8\|Q5F3R8_CHICK | Q5F3R8 | RAB11B | Uncharacterized protein OS=Gallus gallus OX=9031 GN=RAB11B PE=2 SV=1 | CHICK | 2 |
| 26.24999881 | 26.24999881 | 24.02999997 | tr\|Q5F411\|Q5F411_CHICK | Q5F411 | CCT5 | CCT-epsilon OS=Gallus gallus OX=9031 GN=CCT5 PE=2 SV=1 | CHICK | 16 |
| 25.6099999 | 21.68000042 | 19.63 | tr\|Q5F424\|Q5F424_CHICK | Q5F424 | CCT2 | CCT-beta OS=Gallus gallus OX=9031 GN=CCT2 PE=2 SV=1 | CHICK | 9 |
| 11.15999967 | 4.958999902 | 4.958999902 | tr\|Q5F436\|Q5F436_CHICK | Q5F436 | RCJMB04_3j20 | Uncharacterized protein OS=Gallus gallus OX=9031 GN=RCJMB04_3j20 PE=2 SV=1 | CHICK | 1 |
| 5.068999901 | 1.843000017 | 1.843000017 | tr\|Q5F491\|Q5F491_CHICK | Q5F491 | DDX3X | RNA helicase OS=Gallus gallus OX=9031 GN=DDX3X PE=2 SV=1 | CHICK | 1 |
| 3.485000134 | 1.970000006 | 1.970000006 | tr\|Q5F4B9\|Q5F4B9_CHICK | Q5F4B9 | ABCD3 | Uncharacterized protein OS=Gallus gallus OX=9031 GN=ABCD3 PE=2 SV=1 | CHICK | 1 |
| 22.51999974 | 22.51999974 | 22.51999974 | tr\|Q5ZHW8\|Q5ZHW8_CHICK | Q5ZHW8 | RPS14 | Uncharacterized protein OS=Gallus gallus OX=9031 GN=RPS14 PE=2 SV=1 | CHICK | 2 |
| 9.578999877 | 9.578999877 | 9.578999877 | sp\|Q5ZHZ0\|DX39B_CHICK | Q5ZHZ0 | DDX39B | Spliceosome RNA helicase DDX39B OS=Gallus gallus OX=9031 GN=DDX39B PE=2 SV=1 | CHICK | 4 |
| 28.13000083 | 24.06000048 | 21.07000053 | tr\|Q5ZI76\|Q5ZI76_CHICK | Q5ZI76 | RCJMB04_29j22 | C-1-tetrahydrofolate synthase, cytoplasmic OS=Gallus gallus OX=9031 GN=RCJMB04_29j22 PE=2 SV=1 | CHICK | 17 |
| 5.666000023 | 3.358000144 | 3.358000144 | sp\|Q5ZIA5\|COPB_CHICK | Q5ZIA5 | COPB1 | Coatomer subunit beta OS=Gallus gallus OX=9031 GN=COPB1 PE=2 SV=1 | CHICK | 2 |
| 11.62 | 11.62 | 10.31000018 | tr\|Q5ZIC4\|Q5ZIC4_CHICK | Q5ZIC4 | RCJMB04_28a17 | RuvB-like helicase OS=Gallus gallus OX=9031 GN=RCJMB04_28a17 PE=2 SV=1 | CHICK | 3 |
| 9.036999941 | 9.036999941 | 7.259000093 | tr\|Q5ZIF4\|Q5ZIF4_CHICK | Q5ZIF4 | SLC25A13 | EF-hand domain-containing protein OS=Gallus gallus OX=9031 GN=SLC25A13 PE=2 SV=1 | CHICK | 4 |
| 7.202000171 | 7.202000171 | 4.70900014 | tr\|Q5ZIQ5\|Q5ZIQ5_CHICK | Q5ZIQ5 | RCJMB04_24e8 | NADH dehydrogenase [ubiquinone] 1 alpha subcomplex subunit 10, mitochondrial OS=Gallus gallus OX=9031 GN=RCJMB04_24e8 PE=2 SV=1 | CHICK | 1 |
| 13.86999935 | 9.249000251 | 9.249000251 | tr\|Q5ZIX2\|Q5ZIX2_CHICK | Q5ZIX2 | RCJMB04_23a9 | Electron transfer flavoprotein subunit alpha OS=Gallus gallus OX=9031 GN=RCJMB04_23a9 PE=2 SV=1 | CHICK | 2 |
| 20.56999952 | 17.55000055 | 14.33999985 | sp\|Q5ZJ54\|TCPZ_CHICK | Q5ZJ54 | CCT6 | T-complex protein 1 subunit zeta OS=Gallus gallus OX=9031 GN=CCT6 PE=1 SV=3 | CHICK | 7 |
| 6.958000362 | 6.5609999 | 5.169000104 | tr\|Q5ZJQ6\|Q5ZJQ6_CHICK | Q5ZJQ6 | DARS | Aspartate--tRNA ligase, cytoplasmic OS=Gallus gallus OX=9031 GN=DARS PE=2 SV=1 | CHICK | 2 |
| 35.92999876 | 35.92999876 | 35.92999876 | tr\|Q5ZKM2\|Q5ZKM2_CHICK | Q5ZKM2 | RCJMB04_10b5 | Elongation factor 1-alpha OS=Gallus gallus OX=9031 GN=RCJMB04_10b5 PE=2 SV=1 | CHICK | 20 |
| 3.145999834 | 3.145999834 | 3.145999834 | tr\|Q5ZKP5\|Q5ZKP5_CHICK | Q5ZKP5 | RCJMB04_9m12 | Cysteine desulfurase, mitochondrial OS=Gallus gallus OX=9031 GN=RCJMB04_9m12 PE=2 SV=1 | CHICK | 1 |
| 1.57600008 | 1.57600008 | 1.57600008 | tr\|Q5ZKR8\|Q5ZKR8_CHICK | Q5ZKR8 | MCM6 | DNA replication licensing factor MCM6 OS=Gallus gallus OX=9031 GN=MCM6 PE=2 SV=1 | CHICK | 1 |
| 8.997000009 | 5.655999854 | 2.827999927 | tr\|Q5ZKX2\|Q5ZKX2_CHICK | Q5ZKX2 | RCJMB04_8n6 | AAA domain-containing protein OS=Gallus gallus OX=9031 GN=RCJMB04_8n6 PE=2 SV=1 | CHICK | 1 |
| 17.10000038 | 12.21999973 | 12.21999973 | sp\|Q5ZL72\|CH60_CHICK | Q5ZL72 | HSPD1 | 60 kDa heat shock protein, mitochondrial OS=Gallus gallus OX=9031 GN=HSPD1 PE=1 SV=1 | CHICK | 5 |
| 9.735000134 | 4.425000027 | 2.434 | tr\|Q5ZL82\|Q5ZL82_CHICK | Q5ZL82 | RCJMB04_7e11 | Isocitrate dehydrogenase [NADP] OS=Gallus gallus OX=9031 GN=RCJMB04_7e11 PE=2 SV=1 | NADP | 1 |
| 36.23999953 | 36.23999953 | 33.55999887 | tr\|Q5ZLG7\|Q5ZLG7_CHICK | Q5ZLG7 | SLC25A6 | ADP/ATP translocase OS=Gallus gallus OX=9031 GN=SLC25A6 PE=2 SV=1 | CHICK | 11 |
| 4.295000061 | 1.651999913 | 1.651999913 | tr\|Q5ZLU4\|Q5ZLU4_CHICK | Q5ZLU4 | RCJMB04_4n15 | 26S proteasome non-ATPase regulatory subunit 2 OS=Gallus gallus OX=9031 GN=RCJMB04_4n15 PE=2 SV=1 | CHICK | 1 |
| 14.44000006 | 10.15999988 | 6.983999908 | tr\|Q5ZLX7\|Q5ZLX7_CHICK | Q5ZLX7 | RCJMB04_4h24 | ClpX-type ZB domain-containing protein OS=Gallus gallus OX=9031 GN=RCJMB04_4h24 PE=2 SV=1 | CHICK | 3 |
| 15.81999958 | 7.761000097 | 7.761000097 | tr\|Q5ZLZ0\|Q5ZLZ0_CHICK | Q5ZLZ0 | RCJMB04_4e21 | Phosphate carrier protein, mitochondrial OS=Gallus gallus OX=9031 GN=RCJMB04_4e21 PE=2 SV=1 | CHICK | 3 |
| 8.382999897 | 8.382999897 | 8.382999897 | tr\|Q5ZLZ6\|Q5ZLZ6_CHICK | Q5ZLZ6 | SLC16A1 | Monocarboxylate transporter 1 OS=Gallus gallus OX=9031 GN=SLC16A1 PE=2 SV=1 | CHICK | 5 |
| 7.215999812 | 7.215999812 | 7.215999812 | tr\|Q5ZM05\|Q5ZM05_CHICK | Q5ZM05 | RCJMB04_3p21 | 14_3_3 domain-containing protein (Fragment) OS=Gallus gallus OX=9031 GN=RCJMB04_3p21 PE=2 SV=1 | CHICK | 1 |
| 8.347000182 | 3.691999987 | 3.691999987 | tr\|Q5ZM62\|Q5ZM62_CHICK | Q5ZM62 | ABCF2 | Uncharacterized protein OS=Gallus gallus OX=9031 GN=ABCF2 PE=2 SV=1 | CHICK | 2 |
| 2.524000034 | 2.524000034 | 2.524000034 | sp\|Q5ZM72\|FACR1_CHICK | Q5ZM72 | FAR1 | Fatty acyl-CoA reductase 1 OS=Gallus gallus OX=9031 GN=FAR1 PE=2 SV=1 | CHICK | 1 |
| 32.03999996 | 10.49999967 | 6.077000126 | tr\|Q5ZMA0\|Q5ZMA0_CHICK | Q5ZMA0 |  | Uncharacterized protein OS=Gallus gallus OX=9031 GN=RCJMB04_2m6 PE=2 SV=1 | CHICK | 1 |
| 3.13199982 | 3.13199982 | 3.13199982 | tr\|Q5ZMC1\|Q5ZMC1_CHICK | Q5ZMC1 | RP11-529K1.3 | RNA helicase OS=Gallus gallus OX=9031 GN=RP11-529K1.3 PE=2 SV=1 | CHICK | 1 |
| 38.35000098 | 36.55999899 | 30.46999872 | tr\|Q5ZMG9\|Q5ZMG9_CHICK | Q5ZMG9 | TCP1 | CCT-alpha OS=Gallus gallus OX=9031 GN=TCP1 PE=2 SV=1 | CHICK | 19 |
| 3.06599997 | 0.68140002 | 0.68140002 | tr\|Q5ZMN9\|Q5ZMN9_CHICK | Q5ZMN9 | RCJMB04_1i14 | DNA-directed RNA polymerase subunit beta OS=Gallus gallus OX=9031 GN=RCJMB04_1i14 PE=2 SV=1 | CHICK | 1 |
| 23.26000035 | 23.26000035 | 23.26000035 | tr\|Q6BDR9\|Q6BDR9_CHICK | Q6BDR9 | Hsp25 | Heat shock protein 25 OS=Gallus gallus OX=9031 GN=Hsp25 PE=2 SV=1 | CHICK | 4 |
| 7.338999957 | 7.338999957 | 5.045999959 | tr\|Q6EE30\|Q6EE30_CHICK | Q6EE30 | -- | Eukaryotic translation elongation factor 1 OS=Gallus gallus OX=9031 PE=2 SV=1 | CHICK | 2 |
| 22.25999981 | 15.33000022 | 15.33000022 | sp\|Q6EE31\|TCPQ_CHICK | Q6EE31 | CCT8 | T-complex protein 1 subunit theta OS=Gallus gallus OX=9031 GN=CCT8 PE=1 SV=3 | CHICK | 7 |
| 7.900000364 | 6.165999919 | 5.009999871 | tr\|Q6WNG8\|Q6WNG8_CHICK | Q6WNG8 | HNRNPH2 | Heterogeneous nuclear ribonucleoprotein H1-like protein OS=Gallus gallus OX=9031 GN=HNRNPH2 PE=2 SV=1 | CHICK | 2 |
| 7.613000274 | 3.367000073 | 3.367000073 | tr\|Q71SG4\|Q71SG4_CHICK | Q71SG4 | CRMP1A | Collapsin response mediator protein-1A OS=Gallus gallus OX=9031 GN=CRMP1A PE=2 SV=1 | CHICK | 2 |
| 32.64999986 | 27.43999958 | 23.97000045 | tr\|Q7SX63\|Q7SX63_CHICK | Q7SX63 | HSP70 | Heat shock protein 70 OS=Gallus gallus OX=9031 GN=HSP70 PE=3 SV=1 | CHICK | 27 |
| 6.752999872 | 1.169000007 | 1.169000007 | tr\|Q8AYP9\|Q8AYP9_CHICK | Q8AYP9 | pfk | ATP-dependent 6-phosphofructokinase OS=Gallus gallus OX=9031 GN=pfk PE=2 SV=1 | CHICK | 1 |
| 6.780000031 | 5.085000023 | 5.085000023 | tr\|Q8QH01\|Q8QH01_CHICK | Q8QH01 | FMO3 | Dimethylaniline monooxygenase [N-oxide-forming] OS=Gallus gallus OX=9031 GN=FMO3 PE=2 SV=1 | N-oxide-forming | 2 |
| 15.02999961 | 10.11999995 | 7.975000143 | sp\|Q90593\|BIP_CHICK | Q90593 | HSPA5 | Endoplasmic reticulum chaperone BiP OS=Gallus gallus OX=9031 GN=HSPA5 PE=1 SV=1 | CHICK | 5 |
| 13.86999935 | 2.240999974 | 2.240999974 | tr\|Q90XD9\|Q90XD9_CHICK | Q90XD9 | -- | Staphylococcal nuclease domain-containing protein 1 (Fragment) OS=Gallus gallus OX=9031 PE=2 SV=1 | CHICK | 1 |
| 85.35000086 | 85.35000086 | 54.78000045 | tr\|Q91021\|Q91021_CHICK | Q91021 | -- | Uncharacterized protein (Fragment) OS=Gallus gallus OX=9031 PE=2 SV=1 | CHICK | 6 |
| 31.47999942 | 15.73999971 | 10.64999998 | sp\|Q98932\|RAB5C_CHICK | Q98932 | RAB5C | Ras-related protein Rab-5C OS=Gallus gallus OX=9031 GN=RAB5C PE=1 SV=1 | CHICK | 2 |
| 12.21000031 | 12.21000031 | 12.21000031 | sp\|Q9DEA3\|PCNA_CHICK | Q9DEA3 | PCNA | Proliferating cell nuclear antigen OS=Gallus gallus OX=9031 GN=PCNA PE=1 SV=1 | CHICK | 2 |
| 13.80999982 | 13.80999982 | 11.19000018 | tr\|Q9I8D6\|Q9I8D6_CHICK | Q9I8D6 | tcp-1 delta | T-complex protein 1 subunit delta OS=Gallus gallus OX=9031 GN=tcp-1 delta PE=2 SV=1 | CHICK | 5 |
| 29.96000051 | 9.717000276 | 9.717000276 | tr\|Q9PTD6\|Q9PTD6_CHICK | Q9PTD6 | -- | 40S ribosomal protein S6 (Fragment) OS=Gallus gallus OX=9031 PE=2 SV=1 | CHICK | 3 |
| 12.3300001 | 6.848999858 | 6.848999858 | tr\|R4GGJ0\|R4GGJ0_CHICK | R4GGJ0 | RPS16 | Uncharacterized protein OS=Gallus gallus OX=9031 GN=RPS16 PE=3 SV=1 | CHICK | 1 |
| 4.120999947 | 1.006999984 | 1.006999984 | tr\|R4GHW9\|R4GHW9_CHICK | R4GHW9 | EPB41 | Band 4.1 OS=Gallus gallus OX=9031 GN=EPB41 PE=4 SV=2 | CHICK | 1 |
| 2.795000002 | 0.776399998 | 0.776399998 | tr\|W6RT25\|W6RT25_CHICK | W6RT25 | ABCB1 | Multidrug resistance protein 1 OS=Gallus gallus OX=9031 GN=ABCB1 PE=2 SV=1 | CHICK | 1 |
| 17.57999957 | 13.94000053 | 13.94000053 | tr\|Z4YJB8\|Z4YJB8_CHICK | Z4YJB8 | DSTN | Actin-depolymerizing factor OS=Gallus gallus OX=9031 GN=DSTN PE=3 SV=2 | CHICK | 2 |
