# Supplementary material for: Unveiling the role of hexon-associated host proteins in fowl adenovirus serotype 4 replication
Source: Front Vet Sci. 2025 Jun 3;12:1562872. doi: 10.3389/fvets.2025.1562872 (PMC12170584; doi:10.3389/fvets.2025.1562872)
Supplement: Supplementary file 6 [file Table_1.docx]

Table 1

Primers used in this study

| Primers Sequence(5‘ 3') |
| --- |
| Hexon-R 5’TACCGGACTCAGATCTCGAGATGGCGGCCCTCACGCCC3’  Hexon-F 5’GTCAGATCCCATGGATCCCACGGCGTTGCCTGTGGC3’  CCT5-R 5’CTGTACAAGGGCGGTTCATCGGCCATGGGGACGCTG 3’  CCT5-F 5’CTGTACAAGGGCGGTTCATCGGCCATGGGGACGCTG 3’  siCCT5-1 5‘-GGAACAGGCTGAACAATTACT-3’  siCCT5-2 5‘-GCTGTGAATGCTGTACTGACA-3’  siCCT5-3 5‘-GGGAGTGATCGTGGATAAAGA-3’  Hexon mRNA --R 5'-GAACATCCCTTGGGCCCA-3'，  Hexon mRNA --F 5'-GAACATCCCTTGGGCCCA-3'.  β-actin-R 5'-CTCTATCCTGGCCTCCCTGT-3',  β-actin-F 5'-GCTGACACCTTCACCATTCC-3' |
