# Supplementary material for: Unveiling the role of hexon-associated host proteins in fowl adenovirus serotype 4 replication
Source: Front Vet Sci. 2025 Jun 3;12:1562872. doi: 10.3389/fvets.2025.1562872 (PMC12170584; doi:10.3389/fvets.2025.1562872)
Supplement: Supplementary file 7 [file Table_2.docx]

Table 2

| Uniprot ID | Protein name | Gene | Protein function |
| --- | --- | --- | --- |
| F1NFJ0 | Minichromosome Maintenance Complex Component 3 | MCM3 | DNA replication |
| Q5ZKR8 | Minichromosome Maintenance Complex Component 6 | MCM6 | DNA replication |
| P0CB50 | peroxiredoxin-1 | PRDX1 | NF-kappa-B regulation |
| E1BY89 | 60S ribosomal protein L23 | RPL23 | Translation |
| A0A1D5P3B1 | 60S ribosomal protein L11 | RPL11 | Translation |
| A0A1D5NVI1 | 60S ribosomal protein L9 | RPL9 | Translation |
| E1BU66 | 60S ribosomal protein L38 | RPL38 | Translation |
| Q5ZKX2 | Proteasome 26S Subunit, ATPase 5 | PSMC6 | Proteasomesubunit |
| Q5ZLU4 | 26S proteasome non-ATPase regulatory subunit 2 | PSMD2 | Proteasomesubunit |
| R4GGJ0 | Ribosomal Protein S16 | RPS16 | Viral mRNA translation |
| A0A1D5NZ06 | Ribosomal Protein S27 | RPS27 | Viral mRNA translation |
| F1N9U0 | pre-mRNA-processing factor 6 | PRPF6 | pre-mRNA processing |
| F1NV33 | DNA mismatch repair protein Msh2 | MSH2 | DNA repair protein |

Hexon–host interacting proteins in chicken cells (selected proteins).
